# Effect of egg production dynamics on the functional response of two parasitoids

**DOI:** 10.1371/journal.pone.0283916

**Published:** 2024-03-08

**Authors:** María Aguirre, Guillermo Logarzo, Serguei Triapitsyn, Hilda Diaz-Soltero, Stephen Hight, Octavio Augusto Bruzzone

**Affiliations:** 1 Fundación para el Estudio de Especies Invasivas (FuEDEI), Hurlingham, Buenos Aires, Argentina; 2 Department of Entomology, University of California, Riverside, California, United States of America; 3 Animal and Plant Health Inspection Service USDA, San Juan, Puerto Rico; 4 USDA-ARS-CMAVE at Center for Biological Control, Florida A&M University, Tallahassee, Florida, United States of America; 5 Consejo Nacional de Investigaciones Científicas y Técnicas (CONICET), San Carlos de Bariloche, Río Negro, Argentina; 6 Instituto Nacional de Tecnología Agropecuaria (INTA), Estación Experimental Agropecuaria Bariloche, San Carlos de Bariloche, Río Negro, Argentina; Universita degli Studi della Basilicata, ITALY

## Abstract

Functional response describes the number of hosts attacked by a parasitoid in relation to host densities and plays an important role by connecting behavioral-level processes with community-level processes. Most functional response studies were carried out using simple experimental designs where the insects were confined to a plain and small arena with different host densities during a fixed period of time. With these designs, other factors that might affect the functional response of parasitoids were not analyzed, such as fecundity, age, and experience. We proposed a series of latent-variables Markovian models that comprised an integrated approach of functional response and egg production models to estimate the realized lifetime reproductive success of parasitoids. As a case study, we used the parasitoids *Anagyrus cachamai* and *A*. *lapachosus* (Hymenoptera: Encyrtidae), two candidate agents for neoclassical biocontrol of the Puerto Rican cactus pest mealybug, *Hypogeococcus* sp. (Hemiptera: Pseudococcidae). The tested species were assessed according to their physiology and prior experience. We estimated the number of mature eggs after emergence, egg production on the first day, egg production rate, the proportion of eggs resorbed, egg resorption threshold, and egg storage capacity. *Anagyrus cachamai* and *A*. *lapachosus* both presented a type III functional response. However, the two parasitoids behaved differently; for *A*. *cachamai*, the number of parasitized hosts decreased with female age and depended on the number of mature eggs that were available for oviposition, whereas *A*. *lapachosus* host parasitism increased with female age and was modulated by its daily egg load and previous experience. The methodology presented may have large applicability in pest control, invasive species management, and conservation biology, as it has the potential to increase our understanding of the reproductive biology of a wide variety of species, ultimately leading to improved management strategies.

## Introduction

Functional response is one of the most commonly used mathematical frameworks to describe and estimate the number of hosts attacked by a parasitoid in relation to host densities [1, 2], allowing the connection of behavioral-level processes with community-level processes. Identifying a parasitoid’s response to changes in host density is central to any description of parasitism because the number of hosts attacked determines development, reproduction, and survival of the parasitoid [3]. This approach is used not only for host-parasitoid interactions, but also to represent consumer-resource interactions in a general sense [4]. Applications of this mathematical framework are found in studies on pest control, invasive species management, and conservation biology [5].

Despite the abundance of functional response models [6], the most frequently used are Holling’s type II and type III [1], describing a hyperbolic saturating curve and a sigmoid curve, respectively. Development of both functional response models requires only two parameters: *a*, attack rate, and *H*, handling time. Attack rate represents the efficiency of a parasitoid in locating hosts via different “areas of discovery” measured in various habitat complexities or experimental arena sizes, according to Rogers [7], or as Holling [1] expresses, efficiency in locating hosts, measured as the proportion of hosts found per time unit. Handling time, *H*, is the time that a parasitoid spends manipulating its hosts. According to Holling [1], its inverse is the asymptotic value of the functional response curve. Type III functional response curves represent situations where a parasitoid switches between two or more host species due to host availability [1] or learning [8]. These curves can also indicate improved efficiency through learning even when only one host is present [9]. Under host switching, a parasitoid’s access to the host is lower at low host density and higher at high host density. This is because the consumer tends to attack the alternative host more when host density is low, resulting in a low attack rate. However, as host density increases, a parasitoid’s attack rate grows more than proportionally due to increased host availability and encounters. In the case of learning, attack rate increases with higher host density. However, there is a constraint on the rate at which hosts can be consumed due to the time required for handling. As the parasitoid becomes saturated with the host, acceleration of the attack rate’s growth diminishes. This causes the functional response curve, known as Holling’s type III functional response, to approach an upper asymptote and exhibit the characteristic S-shape. In simple terms, attack rate reflects the space a parasitoid seeks per unit of time, while handling time is associated with host processing. The S-shaped curve captures both the initial slow increase and the subsequent saturation effect of a parasitoid’s attack rate in response to fluctuations in host density. Functional response models assume continuous foraging by individuals, along with stationary behavioral and physiological processes, when actually a plethora of biological processes are included under these two parameters [10, 11].

Most parasitoid functional response studies are carried out using experimental designs where the insects are confined to a small arena with different host densities during a fixed time period ranging from 1 to 48 hours [12–17]. These experimental designs ignore factors related to parasitoid behavior that affect the functional response, such as fecundity, age, and experience of the wasp [18–20]. The basic Holling’s models do not consider the effect of age or the physiological status of the consumer. However, Varone et al. [21] studied the functional response of the larval parasitoid *Campoletis grioti* (Blanchard) (Hymenoptera: Ichneumonidae) over the entirety of the female’s lifetime. They found that the parasitoid’s attack rate and handling time fluctuated throughout the female’s lifespan, which was dictated by her daily supply of mature eggs.

Parasitoid wasps exhibit a wide spectrum of reproductive strategies that lead to variation in egg production dynamics. Egg load varies throughout the female’s lifespan, responding to both individual physiological and environmental factors [22–26]. In this context, the number of eggs that a female lays during her lifetime is determined by the number of hosts that the female encounters, the number of mature eggs over the female’s life span, and the behavior affecting the oviposition rate [27]. Parasitoids commonly face one of two scenarios. One scenario involves having more mature eggs available for laying than there are oviposition opportunities, while the other scenario entails having more oviposition opportunities than there are mature eggs available for laying; these situations are denoted as *host limitation* (or *time limitation*) and *egg limitation*, respectively [28, 29]. When assessing the quality or availability of a host, females tend to exhibit higher selectivity when their egg supply is limited, but their selectivity diminishes when they are constrained by time [30]. Since egg production is costly, selection should favor production strategies in which parasitoid females do not die before exhausting their egg complement or run out of eggs before all available hosts are used [31]. However, it is impossible to guarantee a perfect match between the presence of mature eggs and host availability due to unpredictable fluctuations in reproductive opportunities, such as host availability, the presence of predators, and weather conditions [32].

Understanding ovarian dynamics is particularly relevant to describing a parasitoids’ foraging behavior because the physiological status of the ovaries may determine, for example, the duration of the pre-reproductive period and the rate of oviposition. Egg limitation is mediated by oviposition and ovarian production, which in turn is regulated by two processes: egg maturation and egg resorption [29]. A parasitoid’s lifespan impacts the degree to which it becomes constrained by time limitations. In order to improve survival in the field, many parasitoids require a carbohydrate supply, such as nectar or hemipteran honeydew, or they may choose to directly feed on their host [33, 34]. Nonetheless, the fluctuation in nutrient intake resulting from the reliance on external stochastic nutrient sources can introduce significant starvation risks. Egg resorption serves as an insurance against stochasticity, although it is regarded as a “last-resort” strategy due to the comparatively low energy content of an egg [35, 36]. What sets resorption apart from other nutrient sources is its manageable nature, as the reserves stored in the eggs become easily accessible to the female when they are most needed. However, it takes time and, if it occurs simultaneously in all ovarioles, precludes oviposition [37].

For more than 80 years, researchers have put forth various models to assess whether the realized lifetime reproductive success of adult female parasitoids is constrained by the limited time available for finding oviposition sites [38–42], or by the finite supply of mature eggs [43, 44]. Rosenheim [45] employed models to investigate how stochastic factors affect the evolution of egg limitation among insects. The study revealed that both egg and time limitations shape the reproductive behavior and population dynamics of insects. These findings highlight the necessity of developing models for insect reproduction and population dynamics that consider both egg and time limitations as constraints, rather than focusing solely on one constraint.

In this study, we proposed a series of latent-variables Markovian models. These models integrate functional response and egg production models to estimate the realized lifetime reproductive success of adult parasitoids. As a case study, we examined the parasitoids *Anagyrus cachamai* Triapitsyn, Logarzo & Aguirre and *Anagyrus lapachosus* Triapitsyn, Aguirre & Logarzo (Hymenoptera: Encyrtidae). These parasitoids are promising candidates for the neoclassical biological control of the mealybug *Hypogeococcus* sp. (Hemiptera: Pseudococcidae), a pest of native cacti in Puerto Rico [46–48]. The cactus mealybug poses a threat to Cactaceae in North America and in other islands in the Caribbean and Central America [49, 50]. The pest was formerly identified as *Hypogeococcus pungens* Granara de Willink [51], but now it is acknowledged that *H*. *pungens* is a species complex, and that the Puerto Rican pest is derived from Brazilian cactus-feeding mealybugs [48, 52].

*Anagyrus cachamai* and *A*. *lapachosus*, native to Argentina and Paraguay, are synovigenic wasps that do not engage in host feeding, but differ in their reproductive biology. The egg load at birth of *A*. *lapachosus* females was lower than that of *A*. *cachamai* and they exhibited a different pattern of egg production. In *A*. *lapachosus* there was a positive correlation between female body size and realized fecundity; however, this correlation was not observed in *A*. *cachamai*. The sex ratio of *A*. *lapachosus* was male-biased: 0.7:1, but in *A*. *cachamai* it was 1:1 [47]. Under Puerto Rican quarantine conditions, both parasitoid species are capable of accepting the cactus pest and develop for several generations on this host [46].

Each parasitoid species was evaluated employing a dynamic variant of the most common functional response models (e.g., [1, 53]), which included population parameters related to the parasitoids’ fecundity (egg resorption and daily egg load, limited by egg load capacity and daily egg production), as well as the age of the female parasitoids. Unlike the techniques used in previous classical methods, we worked on the entire parasitoid lifetime and incorporated physiological processes related to egg load into our evaluation of parasitism efficiency. This technique will provide a more accurate estimation between the two tested parasitoid species according to their physiology and prior experiences.

## Materials and methods

The studies were conducted at the Fundación para el Estudio de Especies Invasivas (FuEDEI), situated in Hurlingham, Buenos Aires, Argentina, extending from January 2014 through December 2016. All experiments and insect rearing were conducted in environmental-controlled chambers (25 ± 1°C, 16:8 L:D, 60–80% RH). All observations were performed under a dissecting microscope at 40X.

Black box experiments were carried out to investigate the reproductive success of *A*. *cachamai* and *A*. *lapachosus* throughout their lives. By conducting functional response experiments, the daily number of parasitized nymphs was estimated for each female species observing the number of emerged wasps in relation to initial nymph density provided. The data was analyzed using a Bayesian approach and the Metropolis-Hastings algorithm, combining functional response and egg production models, to select the best explaining models and calculate their parameters. This approach yielded valuable insights into factors like ovary egg load and resorption, all without the need to sacrifice the females.

### Parasitoid rearing

Laboratory experiments were conducted using colonies of *A*. *cachamai* and *A*. *lapachosus* raised at FuEDEI since 2014 following the methodology outlined in Aguirre et al. [47]. Each primary parasitoid species was reared using first instar nymphs belonging to the “Cactaceae host-clade” of *Hypogeococcus* sp. [54], a congener but distinct species from the cacti-mealybug pest found in Puerto Rico [48]. Pure mealybug colonies were reared without parasitoids on clean potted plants of *Cleistocactus baumannii* (Lem.) Lem. (Cactaceae).

Colonies of *A*. *cachamai* and *A*. *lapachosus* were reared in separate rooms. Four mated females of each wasp species were placed in a plastic cage (2 L) with a 6 cm diameter hole cut in the lid and covered with polyester gauze for ventilation. The cage contained a piece of *C*. *baumannii* (20–25 cm long) infested with about 100 nymphs of *Hypogeococcus* sp. “Cactaceae host-clade”. After 72 hours, the four female parasitoids were extracted from the plastic cage, and the nymphs exposed to the parasitoids were subsequently observed every three days. After the first parasitoid pupa was detected, daily monitoring started, and all parasitoid pupae found were relocated to a Petri dish (1.5 cm high x 5.5 cm diameter), which was covered with plastic food wrap to prevent wasp escape following emergence. Using this process, the wasps’ age, feeding conditions, and mating were controlled. As parasitoids emerged, they were placed in a new Petri dish of equal dimensions with a squashed drop of honey on the bottom, and covered with clear plastic food wrap, to be used either for rearing or experimental purposes. Female parasitoids used in the experiments were 24 hours old, and provided with food, mated, and had no prior oviposition experience. Throughout the paper, mention of *Hypogeococcus* sp. nymphs exposed to female parasitoids refers to the first instar nymphs of *Hypogeococcus* sp. “Cactaceae host-clade” on 20–25 cm long segments of *C*. *baumannii*.

### Functional response experiments

To assess the functional response of the parasitoids *A*. *cachamai* and *A*. *lapachosus*, a similar daily density of non-parasitized *Hypogeococcus* sp. nymphs was subjected to exposure by a female parasitoid beginning 24 hours after her emergence and continuing until her natural demise [21]. The daily number of parasitized nymphs was estimated for each female by documenting the number of emerged parasitoids relative to the initial nymph density offered. Six nymph densities (10, 20, 40, 60, 80, and 110) were used with 5 replications per density. Since females of *Hypogeococcus* sp. are ovoviviparous and deposit their nymphs in intermittent pulses with variations in timing and the number of nymphs produced, and nymphs were provided at the same developmental stage (1st instar) and settled on *C*. *baumannii* stems, the daily number of exposed nymphs was not constant (see Table 1 for host number ranges for each density). The densities chosen for this study were determined through a pilot test, in which densities above 80 nymphs resulted in a plateau in the curve depicting the number of nymphs attacked in relation to the provided host density.

**Table 1:** Laboratory results from functional response experiments with females of *Anagyrus cachamai* and *A*. *lapachosus* at different ages (days) throughout their lifespan.

	Day	No. females alive	Mean number of nymphs offered per day (range)	Mean number of nymphs parasitized per day (range)	Expected number of nymphs parasitized (CI)
***Anagyrus cachamai***					
	1	5	13 (11–14)	1 (0–4)	1.45 (1.19–1.71)
	2	5	12 (9–14)	3 (0–11)	1.33 (0.92–1.74)
	3	5	13 (9–15)	2 (0–5)	1.46 (0.99–1.93)
	4	4	12 (10–15)	2 (0–3)	1.37 (0.77–1.97)
	5	2	11 (9–13)	1 (0–2)	1.22 (0.00–4.90)
	6	1	-	-	-
	7	1	-	-	-
	8	0	-	-	-
	1	5	24 (20–27)	2 (0–7)	3.60 (2.73–4.47)
	2	5	21 (19–24)	8 (0–19)	3.02 (2.45–3.59)
	3	5	22 (19–27)	1 (0–6)	3.29 (2.42–4.16)
	4	2	26 (25–26)	1 (0–2)	3.96 (2.43–5.49)
	5	1	-	-	-
	6	0	-	-	-
	1	5	41 (38–45)	3 (0–12)	8.35 (7.21–9.49)
	2	5	40 (37–42)	5 (0–10)	7.90 (7.16–8.64)
	3	4	40 (37–44)	9 (0–26)	7.97 (6.38–9.56)
	4	2	43 (40–46)	6 (3–9)	8.95 (0.00–21.53)
	5	2	38 (37–38)	20 (12–28)	7.19 (5.30–9.08)
	6	2	39 (38–40)	8 (0–15)	7.65 (3.70–11.60)
	7	2	46	13 (3–23)	9.94 (9.94–9.94)
	8	1	-	-	-
	9	1	-	-	-
	10	1	-	-	-
	11	1	-	-	-
	12	0	-	-	-
	1	5	62 (55–66)	11 (3–22)	15.90 (13.67–18.13)
	2	5	62 (60–65)	24 (5–45)	16.12 (15.00–17.24)
	3	4	65 (60–67)	9 (2–21)	11.29 (9.70–12.88)
	4	2	62 (57–66)	8 (0–16)	7.19 (7.19–7.19)
	5	1	-	-	-
	6	0	-	-	-
	1	5	80 (76–85)	20 (0–66)	24.28 (22.38–26.18)
	2	5	80 (75–86)	20 (0–41)	17.41 (16.83–17.99)
	3	5	79 (75–87)	6 (0–15)	7.55 (7.55–7.55)
	4	1	-	-	-
	5	1	-	-	-
	6	1	-	-	-
	7	0	-	-	-
	1	5	105 (102–109)	46 (24–50)	36.56 (34.87–38.25)
	2	5	104 (98–109)	31 (0–71)	23.81 (17.16–30.46)
	3	5	100 (92–107)	11 (1–24)	7.55 (7.55–7.55)
	4	2	106 (103–108)	1 (0–1)	7.19
	5	1	-	-	-
	6	1	-	-	-
	7	0	-	-	-
***Anagyrus lapachosus***					
	1	5	14 (13–15)	0 (0–2)	1.13 (0.97–1.29)
	2	5	14 (12–15)	0 (0–1)	1.13 (0.93–1.33)
	3	5	13 (12–16)	2 (0–10)	1.08 (0.77–1.39)
	4	5	14 (13–16)	4 (0–16)	1.20 (0.95–1.45)
	5	5	13 (11–15)	1 (0–2)	1.02 (0.73–1.31)
	6	2	14 (11–16)	1 (0–1)	1.11 (0.00–5.78)
	7	1	-	-	-
	8	1	-	-	-
	9	0	-	-	-
	1	5	24 (22–27)	0 (0–1)	3.28 (2.50–4.06)
	2	5	24 (22–26)	1 (0–3)	3.07 (2.56–3.50)
	3	4	25 (24–26)	2 (0–5)	3.47 (3.07–3.87)
	4	4	25 (23–27)	5 (0–17)	3.48 (2.78–4.18)
	5	3	24 (24–25)	0 (0–1)	3.24 (2.89–3.59)
	6	3	23 (20–27)	1 (0–1)	2.95 (0.76–5.14)
	7	2	23 (19–27)	9 (0–17)	2.99 (0.00–15.03)
	8	2	21 (20–22)	4 (0–8)	2.46 (0.00–5.25)
	9	2	23 (20–25)	6 (0–12)	2.82 (0.00–10.19)
	10	2	23 (21–25)	2 (0–3)	2.93 (0.00–8.95)
	11	1	-	-	-
	12	1	-	-	-
	13	0	-	-	-
	1	5	44 (40–49)	5 (0–24)	9.87 (7.86–11.88)
	2	5	45 (40–50)	5 (0–24)	10.11 (8.16–12.06)
	3	5	46 (40–55)	16 (5–43)	10.67 (7.62–13.72)
	4	5	43 (40–47)	11 (0–31)	9.52 (8.06–10.98)
	5	4	43 (35–48)	0	9.61 (6.03–13.19)
	6	2	46 (42–50)	24 (22–25)	10.73 (0.00–32.56)
	7	2	41 (36–46)	25 (21–28)	8.71 (0.00–33.60)
	8	2	47 (46–47)	23 (0–46)	10.88 (8.09–13.67)
	9	1	-	-	-
	10	0	-	-	-
	1	5	62 (59–65)	8 (0–26)	18.32 (16.31–20.33)
	2	5	64 (62–67)	14 (0–45)	19.49 (18.22–20.76)
	3	3	60 (59–62)	18 (0–37)	17.70 (15.71–19.69)
	4	2	59 (56–61)	26 (11–41)	16.51 (1.33–31.69)
	5	2	66 (65–66)	17 (15–19)	20.30 (16.80–23.80)
	6	2	63 (60–65)	22 (0–43)	18.69 (1.71–35.67)
	7	1	-	-	-
	8	1	-	-	-
	9	1	-	-	-
	10	0	-	-	-
	1	5	82 (76–86)	29 (3–53)	29.93 (26.48–33.38)
	2	5	83 (76–86)	12 (0–35)	17.16 (13.71–20.61)
	3	4	85 (83–86)	18 (3–26)	13.14 (13.14–13.14)
	4	3	83 (81–85)	3 (0–5)	17.26 (17.26–17.26)
	5	0	-	-	-
	1	5	107 (100–110)	27 (3–81)	37.08 (37.08–37.08)
	2	5	107 (100–113)	10 (0–22)	10.01 (10.01–10.01)
	3	5	108 (106–111)	19 (3–50)	13.14 (13.14–13.14)
	4	3	108 (106–111)	23 (0–49)	17.26 (17.26–17.26)
	5	1	-	-	-
	6	1	-	-	-
	7	1	-	-	-
	8	1	-	-	-
	9	0	-	-	-

The mean number of nymphs offered per day, the mean number of parasitized nymphs per day, and the expected number of parasitized nymphs are not presented for those nymph/day density combinations in which fewer than two females survived.

Experiments took place in vented plastic containers similar to the one previously described for rearing parasitoids. To ensure that the daily number of non-parasitized nymphs available to each wasp was similar, the cactus piece with the nymphs exposed to the wasp was removed every 24 hours from the experimental arena and replaced by another piece of cactus with a similar number of nymphs not previously exposed to the parasitoid. Each cactus piece with nymphs exposed to the parasitoid was held individually in a similar plastic rearing cage. The exposed nymphs were checked every three days to record the number of emerged parasitoids until all non-parasitized nymphs completed their development and all wasps had emerged from parasitized nymphs. Since it is not possible to distinguish parasitized from non-parasitized nymphs before mealybugs complete their development, development failure and superparasitism are considered part of the experimental design error. It was assumed that parasitism does not cause differential mortality on the host and that there was no superparasitism.

### Data analyses

#### Description of models

The outcome of the functional response experiments was analyzed using a series of latent-variables Markovian models, summarized in a single equation (I) that combines the functional response equation (*f*(*n*)) from the functional response module, and the egg production equation (*e*(*t*)) from the egg production module, employing the minimum function (∧), based on the Law of Minimum proposed by Liebig [55]:

p(n,t)=f(n)∧e(t)

In this equation, *p*(*n*, *t*) represents the model describing the number of eggs laid by a wasp, *e*(*t*) indicates the number of eggs available at time *t*, and *f*(*n*) represents the number of eggs that can be laid. When the number of eggs available (based on the female’s egg load) exceeds the number of eggs that can be oviposited according to the functional response equation ((*e*(*t*)>*f*(*n*)), then the number of hosts attacked is predicted by the functional response equation, and as a result, the female oviposits *f*(*n*) eggs. However, if the female’s egg load is lower than the number of eggs predicted by the functional response equation (*e*(*t*)<*f*(*n*)), the female oviposits *e*(*t*) eggs, which means that she lays all the available eggs, indicating that the parasitoid is egg-limited. The equation assumes that a female cannot lay more eggs than she possesses, and there is no reason for her to retain mature, unlaid eggs in excess of suitable hosts. The proposed models combine one of the six functional response equations from the functional response module and one of the eight egg production equations from the egg production module. This results in a matrix of 48 possible models, all of which were tested. Below are brief descriptions of both modules.

*Functional response module*. The six equations tested in the functional response module were based on type I, type II, and two type III generalized functional responses [1, 9, 53, 56–58]. For each of the type III generalized functional response equations, an additional version where the female gains experience in the course of her life when interacting with the host was also proposed. See the appendix section in the supporting information for details on the six equations tested (S1 File, Functional response module).

*Egg production module*. Eight egg production equations were proposed and rigorously tested, starting with the simplest equation that assumes unlimited egg production by the female and gradually advanced to more complex models by introducing additional parameters. One of these slightly more complex models considers that the female has a limited stock of eggs that is not replenished. This can be interpreted as assuming the extreme behavior where the female is strictly pro-ovigenic [27], meaning all of its oocytes are mature upon emergence. The remaining six equations describe the behavior of females that exhibit synovigenic traits. Synovigenic [27] refers to insects that continuously produce mature eggs throughout their reproductive lifespan. In this reproductive mode, females can emerge with no mature eggs or with many eggs, continuing to mature them throughout their lifetime. The most complex synovigenic-based model developed includes parameters related to egg resorption and daily egg load, limited by egg load capacity and daily egg production. The eight egg production equations proposed for testing within the egg production module are presented in the appendix section in supporting information (S1 File, Egg production module).

#### Model fitting and selection

We used a fully Bayesian approach and the Metropolis-Hastings algorithm [59, 60] in order to select the best-explaining models (out of the 48 models proposed) and to calculate their parameters. Traditionally, statistical analysis of functional response experiments is carried out by selecting the functional response model by a logistic regression, thereby reducing the problem of differentiating between a hyperbolic curve (type II functional response) and a sigmoid curve (type III functional response). Use of a non-linear regression in a frequentist framework is then recommended to estimate the parameters of the curve [61]. Since this approach is not appropriate for selecting several models that compete with one another, Johnson and Omland [62] proposed the use of the Bayesian system. In this work, selection of models and estimation of parameter distribution was conducted in a Bayesian framework. Results of the analysis enabled us to infer which models and parameters explained the results of the laboratory experiments and their statistical distributions.

The Deviation Information Criterion (DIC) index was used as the decision rule for model selection [63]. Models that presented lower DIC were selected according to Gelman et al. [64], as a balance of the explanatory power (in terms of the likelihood function) and complexity (in terms of the number of parameters). It is necessary to obtain DIC values that have a difference greater than 5 among the different models in order to select one model over other models. If DIC values among the different models are not greater than 5, then model averaging is required following Burnham and Anderson [65]. A total of 200,000 iterations were used to fit the models; the first 100,000 were discarded as a “burn-in” for model selection, and the remaining iterations were used to calculate parameters of each model and the information indexes. The a *priori* distribution of the parameters of the functional response curves were normal distributions with a mean of 0 and a variance equal to 100, or uniform distributions defined between the minimum and the maximum value that each parameter can obtain. In some parameters, such as handling time (*H*), attack rate of the female when she emerges (*b*), the number of mature eggs when a female emerges (*h*_0_), egg resorption threshold (*u*), and egg storage capacity (*C*), the values were restricted to be positive, since negative values would not make biological sense. Since the variables obtained (number of parasitized hosts) are discrete and bounded, the binomial likelihood function was used [64]. Finally, the fitness of each selected model to the data of the experiments was calculated by using the generalized coefficient of determination (GCD) for binary data, according to Cox and Snell [66] and Magee [67]. Analyses were carried out using a Parasitoid-Egg model version 0.02 [68] for the parasitoid model, and PyMC version 2.3.7 for Monte Carlo methods [69] for parameter calculation and fitting.

## Results

Laboratory experiments on the functional response of the parasitoid *A*. *cachamai* revealed a consistent decline in the number of attacked hosts over time, regardless of the number of hosts provided (Table 1). During the initial two days of their lifespan, females displayed peak egg-laying activity. In contrast, *A*. *lapachosus* females exhibited highly variable oviposition patterns that continued until the death of the females, and also depended on the host density offered (Table 1). *Anagyrus cachamai* females lived an average of 4 ± 2 days (range 2–11 days) while *A*. *lapachosus* females lived for 5 ± 3 days (range 2–12 days).

Out of the initial set of 48 models proposed to explain the observed oviposition pattern in the laboratory experiments conducted with the parasitoid species *A*. *cachamai*, four models with very close DIC values were chosen: *C*5 (DIC = 1933.43), *E*5 (DIC = 1932.39), *C*7 (DIC = 1933.38) and *E*7 (DIC = 1935.21) (S1 Table). Their explanatory power in terms of GCD was 0.85 (*C*5), 0.85 (*E*5), 0.85 (*C*7), and 0.86 (*E*7), respectively. The difference in DIC values of the selected models was less than 5, making it impossible to identify a single model.

**Fig 1:**
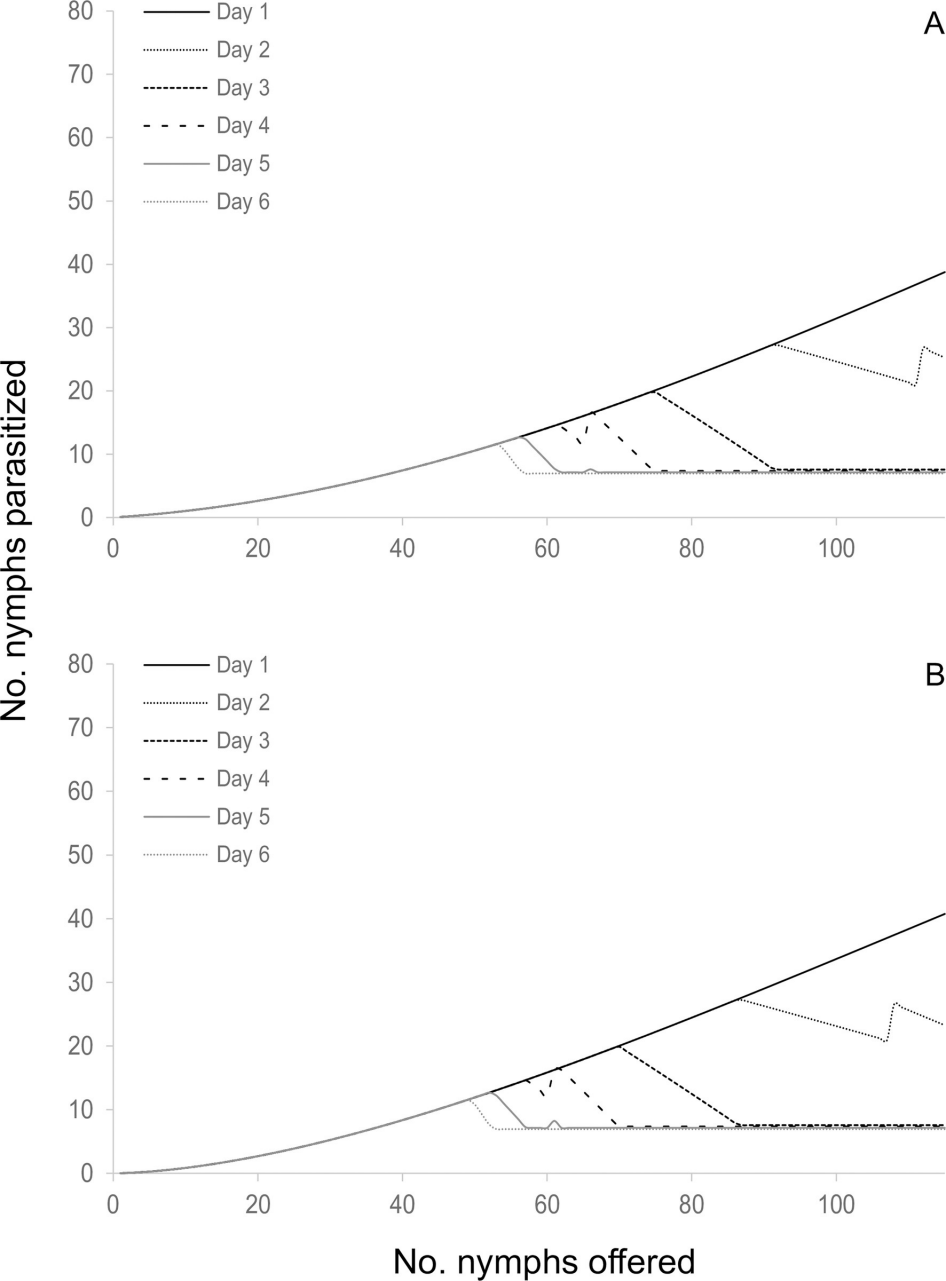
Estimated type III functional response of *Anagyrus cachamai* females at different ages (days) of their average lifespan (1–6 days) considering limited egg production. (A) Equation *C* of functional response module; the attack rate increases linearly with the number of hosts available [56, 57]. (B) Equation *E* of functional response module; the attack rate changes with host densities as *an*^*s*^ [53].

**Fig 2:**
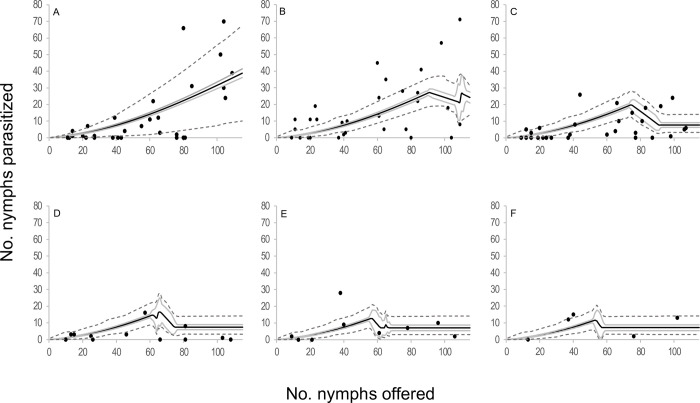
Observed functional response of the parasitoid *Anagyrus cachamai* attacking *Hypogeococcus* sp. nymphs at different ages (days) of their average lifespan. (A-F) Solid line indicates the mean estimation of functional response for model *C*5 at different ages of female lifespan (1–6 days), grey line indicates its credibility interval, and dashed line indicates the *a posteriori* credibility interval for individual measurements. Dark circles are the observed number of emerged parasitoids.

**Fig 3:**
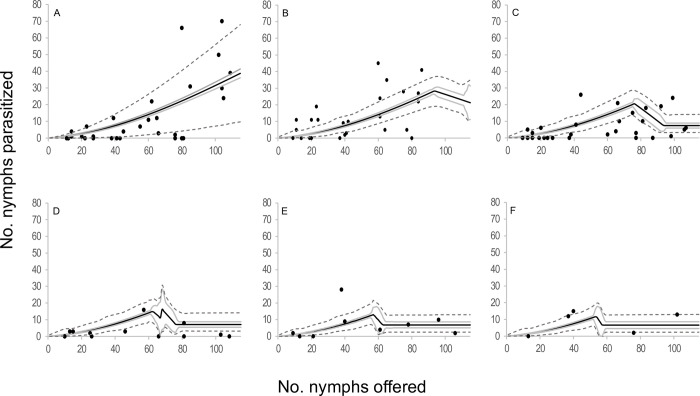
Observed functional response of the parasitoid *Anagyrus cachamai* attacking *Hypogeococcus* sp. nymphs at different ages (days) of their average lifespan. (A-F) Solid line indicates the mean estimation of functional response for model *C*7 at different ages of female lifespan (1–6 days), grey line indicates its credibility interval, and dashed line indicates the *a posteriori* credibility interval for individual measurements. Dark circles are the observed number of emerged parasitoids.

**Fig 4:**
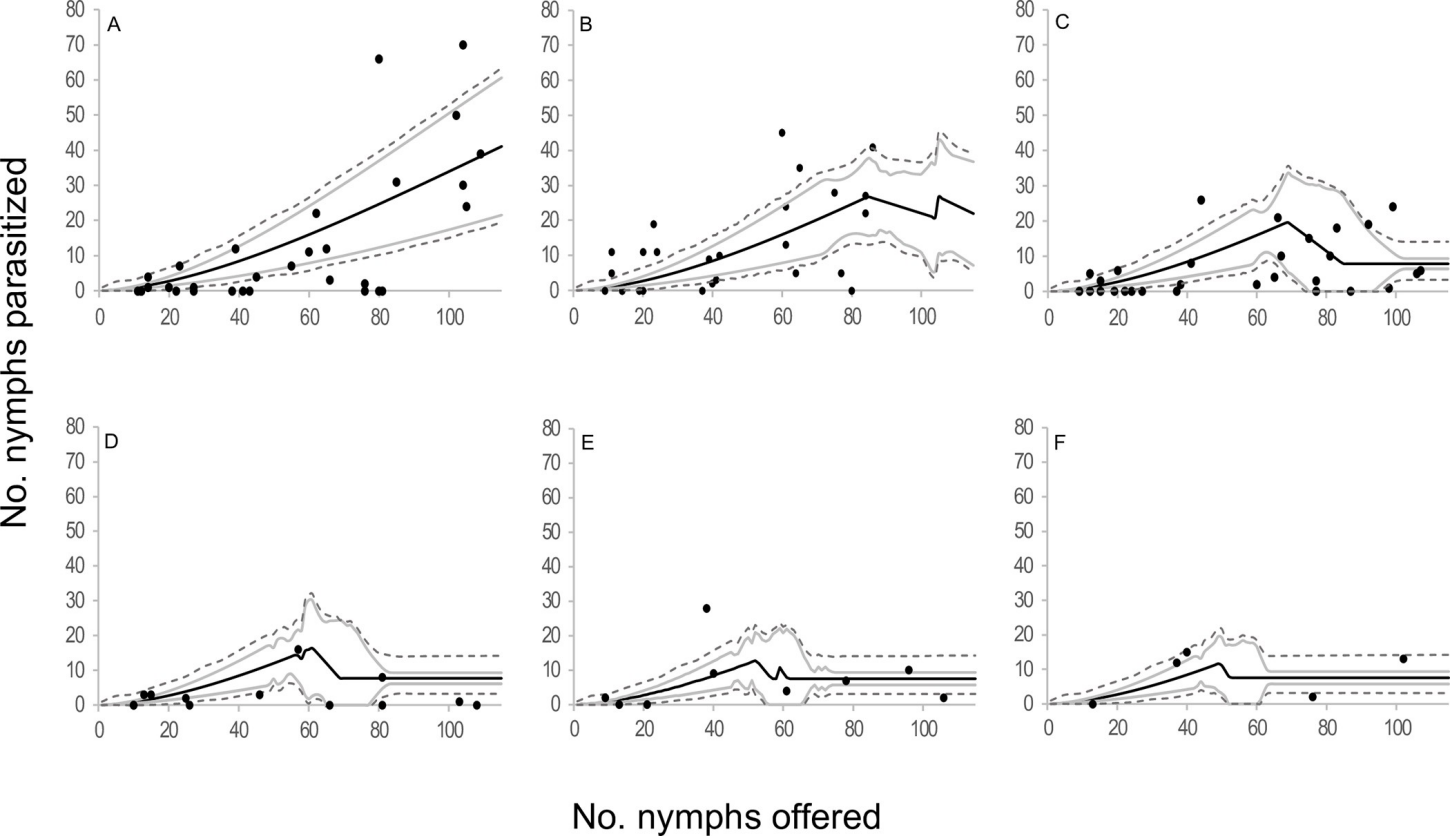
Observed functional response of the parasitoid *Anagyrus cachamai* attacking *Hypogeococcus* sp. nymphs at different ages (days) of their average lifespan. (A-F) Solid line indicates the mean estimation of functional response for model *E5* at different ages of female lifespan (1–6 days), grey line indicates its credibility interval, and dashed line indicates the *a posteriori* credibility interval for individual measurements. Dark circles are the observed number of emerged parasitoids.

**Fig 5:**
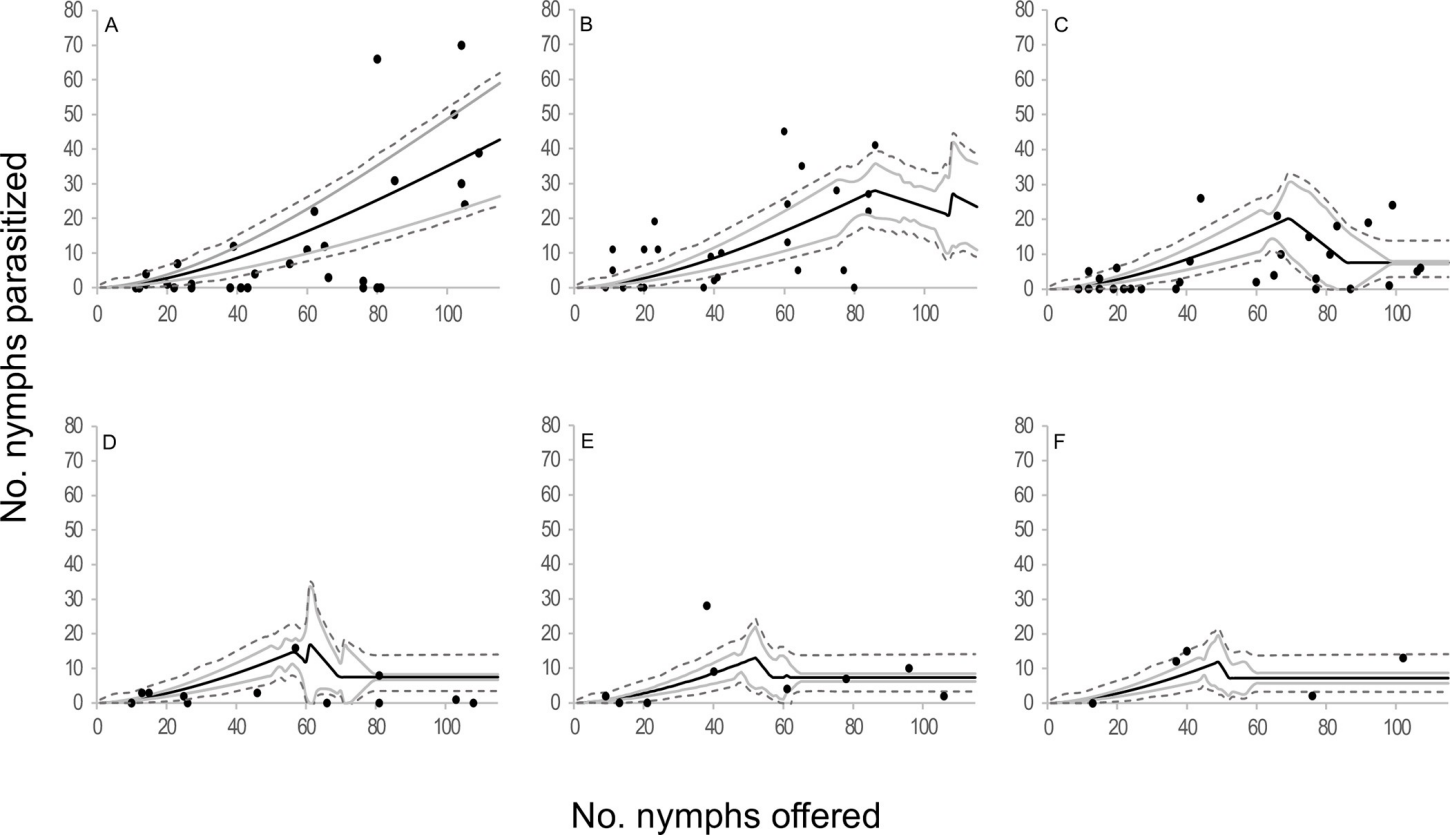
Observed functional response of the parasitoid *Anagyrus cachamai* attacking *Hypogeococcus* sp. nymphs at different ages (days) of their average lifespan. (A-F) Solid line indicates the mean estimation of functional response for model *E7* at different ages of female lifespan (1–6 days), grey line indicates its credibility interval, and dashed line indicates the *a posteriori* credibility interval for individual measurements. Dark circles are the observed number of emerged parasitoids.

**Fig 6:**
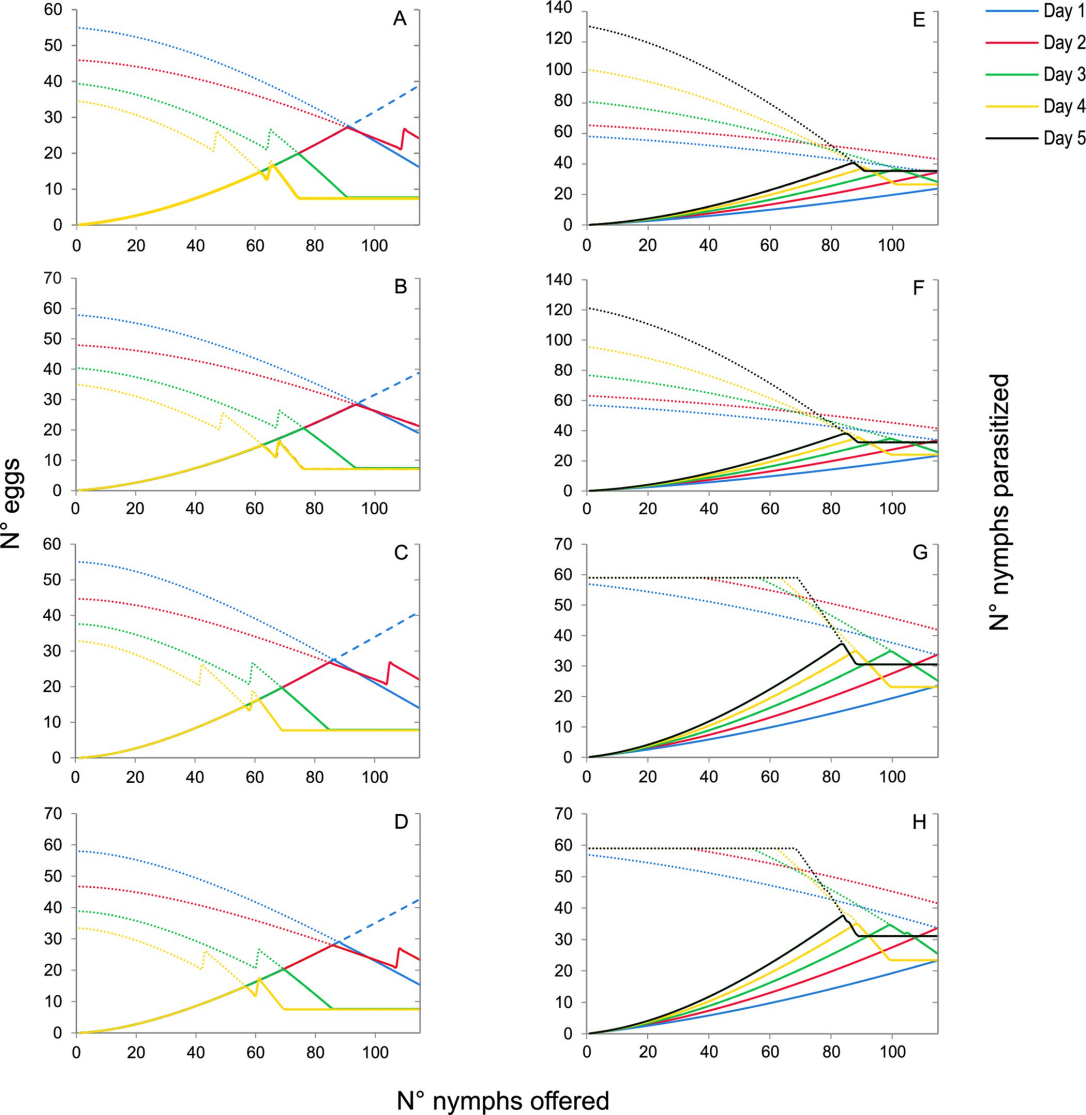
Estimated type III functional response and egg production models selected for the parasitoids *Anagyrus cachamai* (A-D) and *A*. *lapachosus* (E-H) at different ages (days) of their average lifespan. (A) Model *C*5; (B) model *C*7; (C) model *E*5; (D) model *E*7; (E) model *D*4; (F) model *D*5; (G) model *D*6; (H) model *D*7. Dashed lines indicate the mean estimation of functional response models considering limited egg production, dotted lines represent estimated egg availability, and solid lines denote the minimum value between the functional response and egg production functions.

When the components of the four selected models were analyzed in terms of functional response, two type III functional response equations were selected: *C* and *E* (Table 2, Fig 1A and 1B). In those models where equation *C* of the functional response module was selected, the attack rate increased linearly with the number of hosts offered. The emerged female attack rate was 0.073 ± 0.015 days^-1^(*b*), the attack rate change was 0.003 ± 0.000 days^-1^ (*a*), and the handling time was 0.005 ± 0.001 days (*H*). In models where equation *E* of the functional response module was selected, the attack rate changed with host densities as *an*^*s*^, where *a* = 0.018 ± 0.005 days^-1^, *s* = 1.676 ± 0.063 and *H* = 0.005 ± 0.001 days. In reference to the egg production module, two equations were selected; equation 5 and equation 7. *Anagyrus cachamai* females emerged with 56 ± 2 mature eggs (*e*). During the first day of life, a female produced 8 ± 1 eggs (*h*_0_), and the daily egg production rate was 0.972 ± 0.036 days^-1^ (*g*). Eggs that were not used on day t were reabsorbed on day t+1, as long as the accumulated number from one day to the next exceeded 19 ± 1 eggs (*u*). When resorption occurred, the proportion of eggs resorbed was 0.677 ± 0.045. In equation 7, females also exhibited an egg storage capacity of 58 ± 2 eggs (*C*). The four integrated models that were selected are depicted in Figs 2–5 and detailed in S2 Table, as well as S1–S4 Figs. These results indicated that during the initial two days, the number of hosts attacked by *A*. *cachamai* females was predicted by the functional response equation. Beyond the third day, as the females’ egg stock started to deplete, the number of hosts attacked was predicted by the egg production equation (Fig 6A–6D).

**Table 2:** *A posteriori* mean ± standard deviation of the species-specific parameters of the selected models for two parasitoids, *Anagyrus cachamai* and *A*. *lapachosus*, attacking *Hypogeococcus* sp. The values presented are the result of weighing the models selected for *A*. *cachamai* and *A*. *lapachosus* females (see the supporting information for details on the models selected: S1–S3 Tables).

	**Functional response module parameters**													
	**Without female experience**									**With female experience**				
	**FR III (attack rate increased linearly with the available host number [56, 57])**					**FR III (the attack rate changed with host densities as *an***^***s***^ **[53])**				**FR III (attack rate increased linearly with the available host number [56, 57])**				
	Attack rate after emergence at *n* = 0 (*b*)	Attack rate change (*a*)		Handling time (*H*)		Attack rate change (*a*)	Exponent *s* (1+*q*)		Handling time (*H*)	Attack rate after emergence at *n* = 0 (*b*)		Attack rate change (*a*)		Handling time (*H*)
***A*. *cachamai***	0.073±0.015 *d*^-1^	0.003±0.000 *d*^-1^		0.005±0.001 *d*		0.018±0.005 *d*^-1^	1.676±0.063 *d*^-1^		0.005±0.001 *d*	-		-		-
***A*. *lapachosus***	-	-		-		-	-		-	0.109±0.010 *d*^-1^		0.001±0.000 *d*^-1^		0.004±0.001 *d*
	**Egg production module parameters**													
	No. mature eggs after emergence (*e*)		Egg prod. on the first day (*h*_0_)		Egg production rate (*g*)			Proportion of eggs resorbed (*r*)			Egg resorption threshold (*u*)		Egg storage capacity (*C*)	
***A*. *cachamai***	56±2		8±1		0.972±0.036 *d*^-1^			0.677±0.045			19±1		58±2	
***A*. *lapachosus***	57±2		11±1		1.330±0.024 *d*^-1^			0.935±0.028			15±1		59±2	

Parameters reported for the egg production term are averaged considering all the iterations with the functional response type III without female experience in the case of the species *A*. *cachamai* or with experience for *A*. *lapachosus*. On the other hand, the functional response parameters were not mixed because the values and their behavior were slightly different depending on the kind of model selected. Physical units of the calculated parameters: *d* is days, parameters without units are dimensionless.

Models selected for the species *A*. *lapachosus* were *D*4 (DIC = 2974.66), *D*5 (DIC = 2973.60), *D*6 (DIC = 2976.63), and *D*7 (DIC = 2975.19) (S1 Table); and their explanatory power in terms of GCD was 0.86 (*D*4), 0.86 (*D*5), 0.86 (*D*6), and 0.86 (*D*7), respectively. The DIC values of the selected models exhibited a difference of less than 5, precluding the selection of a unique model.

**Fig 7:**
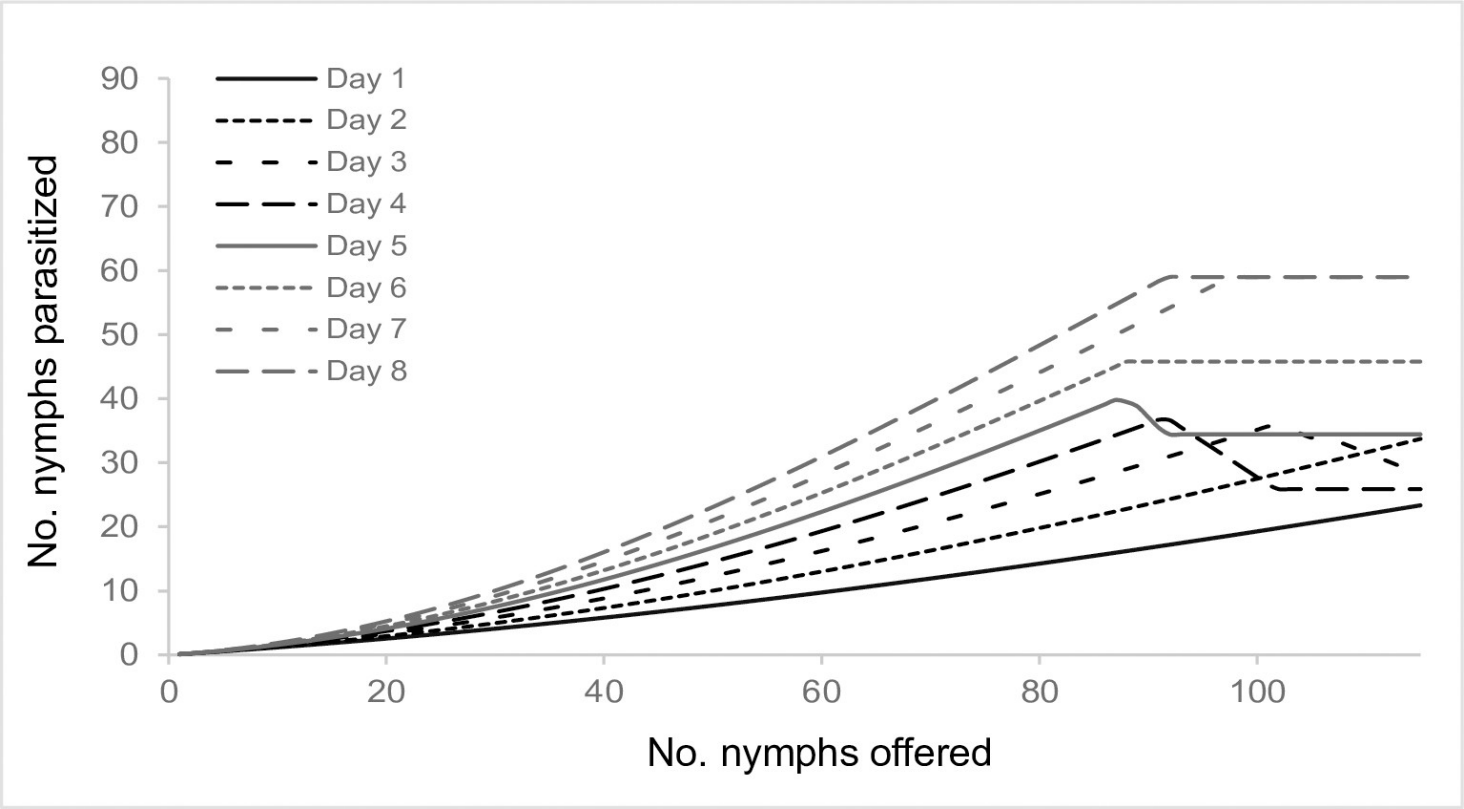
Estimated type III functional response of *Anagyrus lapachosus* females at different ages (days) of their average lifespan (1–8 days) considering limited egg production and female experience. In equation *D* of the functional response module, the female gains experience throughout her life by interacting with the hosts. This is reflected in a daily increase in her attack rate, expressed by the growing steepness of the functional response curve over time.

**Fig 8:**
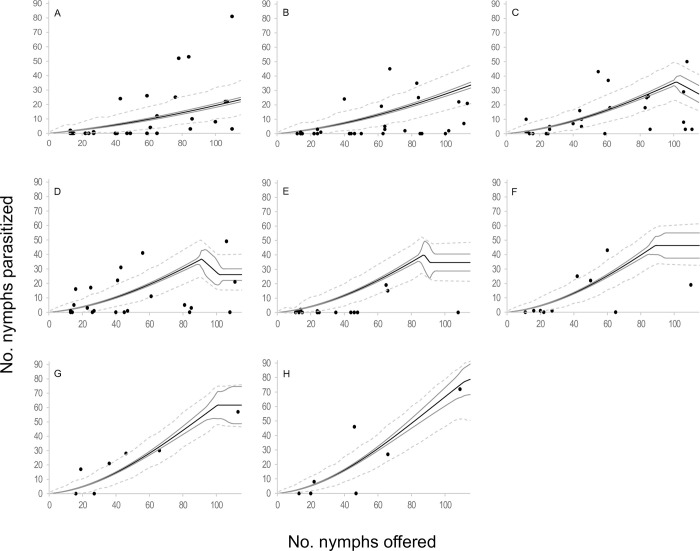
Observed functional response of the parasitoid *Anagyrus lapachosus* attacking *Hypogeococcus* sp. nymphs at different ages (days) of their average lifespan. (A-H) Solid line indicates the mean estimation of functional response for model *D*4 at different ages of female lifespan (1–8 days), grey line indicates its credibility interval, and dashed line indicates the *a posteriori* credibility interval for individual measurements. Dark circles are the observed number of emerged parasitoids.

**Fig 9:**
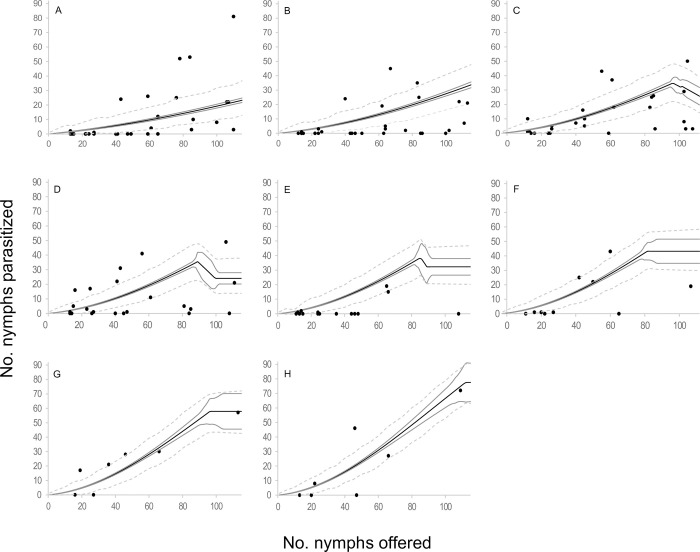
Observed functional response of the parasitoid *Anagyrus lapachosus* attacking *Hypogeococcus* sp. nymphs at different ages (days) of their average lifespan. (A-H) Solid line indicates the mean estimation of functional response for model *D*5 at different ages of female lifespan (1–8 days), grey line indicates its credibility interval, and dashed line indicates the *a posteriori* credibility interval for individual measurements. Dark circles are the observed number of emerged parasitoids.

**Fig 10:**
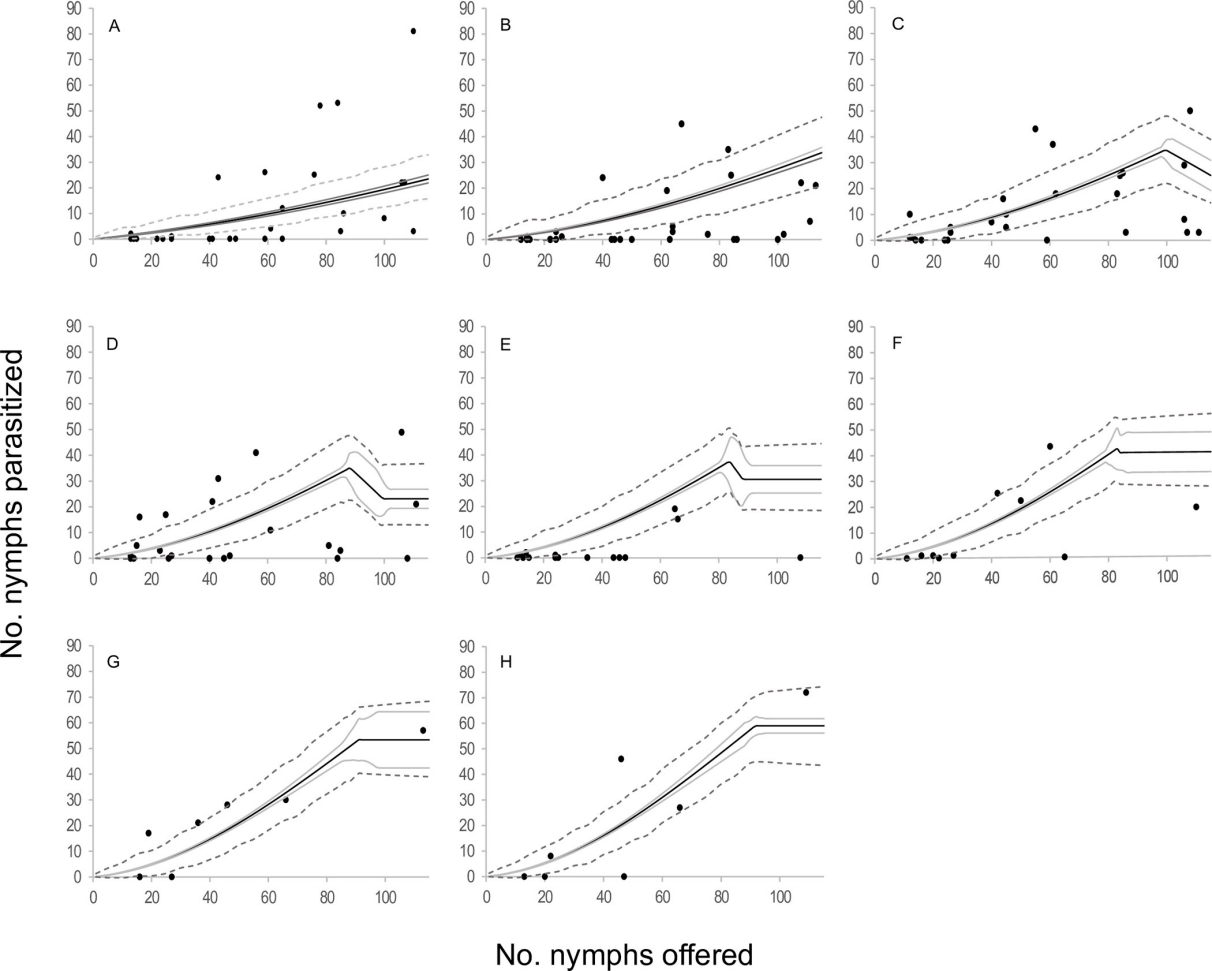
Observed functional response of the parasitoid *Anagyrus lapachosus* attacking *Hypogeococcus* sp. nymphs at different ages (days) of their average lifespan. (A-H) Solid line indicates the mean estimation of functional response for model *D*6 at different ages of female lifespan (1–8 days), grey line indicates its credibility interval, and dashed line indicates the *a posteriori* credibility interval for individual measurements. Dark circles are the observed number of emerged parasitoids.

**Fig 11:**
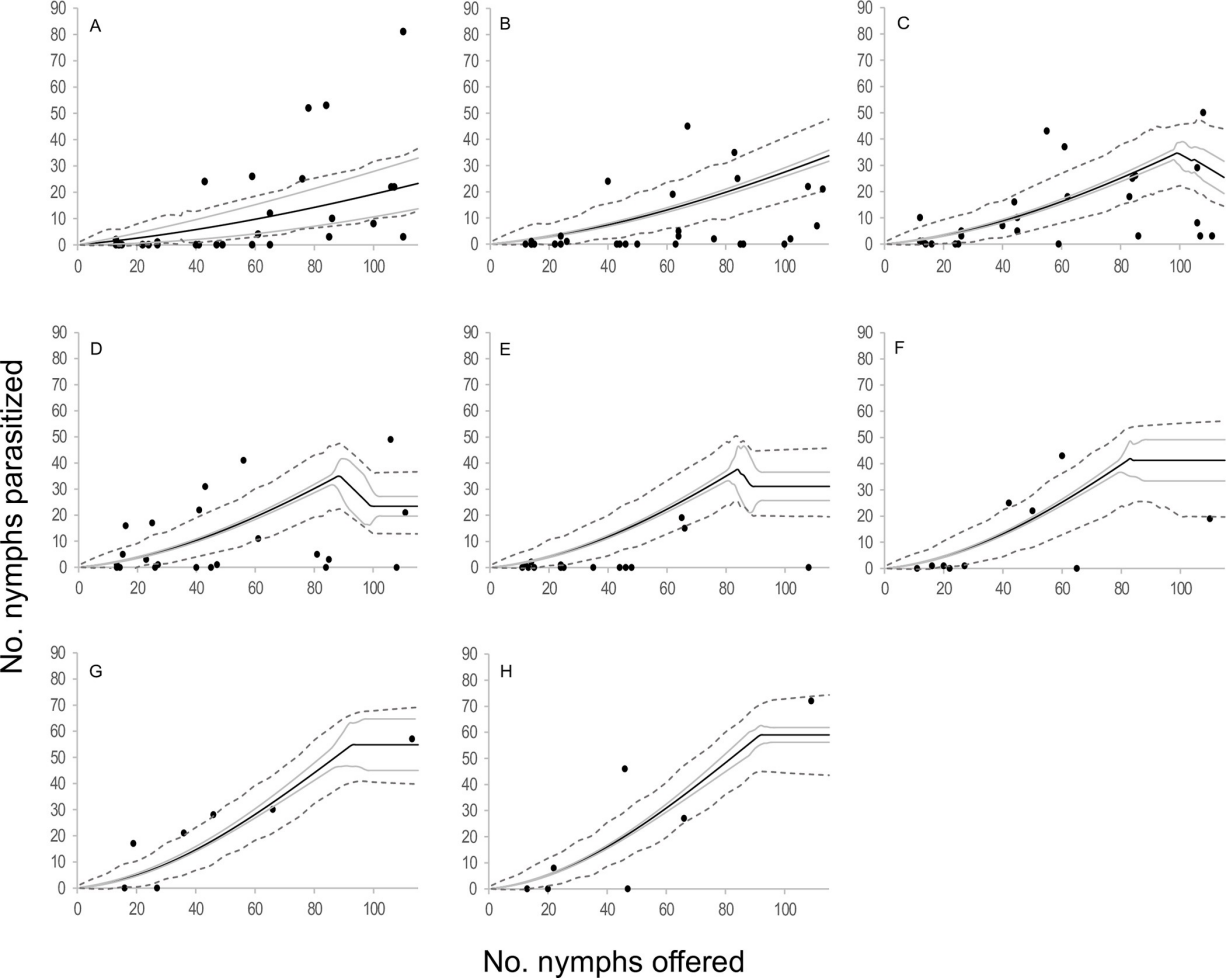
Observed functional response of the parasitoid *Anagyrus lapachosus* attacking *Hypogeococcus* sp. nymphs at different ages (days) of their average lifespan. (A-H) Solid line indicates the mean estimation of functional response for model *D*7 at different ages of female lifespan (1–8 days), grey line indicates its credibility interval, and dashed line indicates the *a posteriori* credibility interval for individual measurements. Dark circles are the observed number of emerged parasitoids.

In the functional response module, the best-explaining models were those that incorporated a type III functional response, as represented by Equation *D*. This response type implies that the attack rate rises with the number of hosts presented during a female’s lifetime, suggesting that females accumulated experience through host interactions over their lifespan. In terms of functional response curve, this means a daily increase in the attack rate of *A*. *lapachosus* females, reflected in the progressively steeper slope of the functional response curve over time (Table 2, Fig 7). The emerging female attack rate was 0.109 ± 0.010 days^-1^ (*b*), the daily attack rate change was 0.001 ± 0.000 days^-1^ (*a*), and the handling time was 0.004 ± 0.001 days (*H*). Regarding the egg production module, four egg production equations were selected: 4, 5, 6, and 7. *Anagyrus lapachosus* females emerged with 57 ± 2 mature eggs (*e*). Post-emergence, each female produced 11 ± 1 eggs (*h*_0_), with a daily egg production rate of 1.330 ± 0.024 days^-1^ (*g*). Eggs not utilized on day t were resorbed the following day in a proportion of 0.935 ± 0.028 (*r*). For equations 5 and 7, resorption occured only if the number of remaining eggs from one day to the next exceeded a threshold of 15 ± 1 eggs (*u*). Finally, for equations 6 and 7, females presented a maximum egg storage capacity of 59 ± 2 eggs (*C*). The models *D*4, *D*5, *D*6, and *D*7 are visually represented in Figs 8–11, and are thoroughly described in S3 Table, as well as in S5–S8 Figs. In this species, according to the selected models, during the first two days, the number of hosts parasitized depended on females’ ability to consume nymphs. After the third day, parasitism was modulated by the females’ egg stock and previous experience (Fig 6E–6H).

## Discussion

Our study aimed to investigate the reproductive success of adult parasitoids using latent-variables Markovian models. We integrated functional response and egg production models, connected by the law of the minimum, into a dynamic model. This approach provided valuable insights into the dynamics of reproductive biology for two candidate species of parasitoids considered for the biological control of a cactus mealybug pest.

The findings, inferred from our proposed models rather than direct observation, align with the following interpretation: the probable cause of diminishing number of hosts parasitized by the species *A*. *cachamai* with increasing females’ age was a reduction in the availability of mature eggs for oviposition. In contrast, for *A*. *lapachosus* females, the daily host parasitism rate was influenced by both daily egg production and the females’ prior experience. The previous experience of *A*. *lapachosus* females became particularly significant in determining the parasitism rate when the egg stock was abundant.

According to Vinson [70], parasitoid females exhibit distinct oviposition behaviors, including host location and evaluation, ovipositor insertion, host acceptance, oviposition, and host marking (either chemical or mechanical, to avoid superparasitism). Although the host finding and attack cycle are innate, experience gained during the oviposition process can enhance a female’s ability to locate and parasitize her host [71]. However, a parasitoid’s “motivation” is another element that is important in describing a parasitoid’s oviposition behavior, and may be influenced by factors such as the onset of hunger, egg load, presence of competitors and predators, as well as environmental changes [70]. This concept is assigned to the category of hidden or latent variables, which cannot be measured directly but only by its correlation with observable behavior [72]. With the models we developed, we explained the oviposition behavior of *A*. *cachamai* and *A*. *lapachosus* against variations in host densities in more detail than with the commonly used classical functional response models [1, 53]. Thanks to the use of Markovian models combined with Bayesian statistics, it was possible to make an accurate description of the ovigeny strategy of *A*. *cachamai* and *A*. *lapachosus*. Furthermore, by studying both species throughout their adult lives, we were able to analyze how age and previous experience with the host influenced their reproductive success.

*Anagyrus cachamai* and *A*. *lapachosus* exhibited a type III functional response. Holling [73] suggested that type III functional responses could be a consequence of parasitoid learning, however, his formulations of this behavior were not permanent. At low host densities, Holling’s model assumed that the contact of the parasitoid with the host would be so rare that the parasitoid could not develop a “search image” for the host. If host density increased, the frequency of contacts would rise and the parasitoid could become more responsive to the specific stimuli of the host. If the parasitoid does not encounter the host for a prolonged period of time, everything learned will be forgotten. The increased foraging behavior exhibited by females after parasitizing the first host may be due to a process known as associative learning [70]. Associative learning is identified as a female’s perception of chemical traces (semiochemicals) and/or physical stimuli of the host (visual or mechanical) after a full oviposition experience, and the parasitoid’s subsequent ability to find, recognize and accept (or reject) other hosts [74]. When a female is rewarded after a full oviposition experience, she learns that her foraging behavior in response to certain plant odors or host cues leads to finding a suitable host. Females of *A*. *cachamai* were “fast learners” after a single oviposition experience, although their response faded at 24 hours (Fig 1A and 1B). In contrast, females of *A*. *lapachosus* were “slow learners”, but they developed a long-term memory, since they showed an increase in their daily attack rate (Fig 7). Learning abilities and memory retention vary among parasitoid species and come at a physiological cost [75, 76]. The different learning skills observed between *A*. *cachamai* and *A*. *lapachosus* females may have resulted from their dissimilar reproductive strategies [76]. Long-term memory may not be needed if oviposition was concentrated during the first days of a female’s lifespan, as in the case with *A*. *cachamai*. On the other hand, it is proposed that, in the short term, egg limitation in parasitoids produces highly saturating functional response curves, similar to the effect of satiation on predators [77]. It was likely that *A*. *cachamai* females, consistently experiencing egg limitation over time, became fast learners after just one oviposition experience, as evidenced by their stronger saturating curve compared to *A*. *lapachosus*.

The selected models confirmed that *A*. *cachamai* and *A*. *lapachosus* are synovigenic, coinciding with the results obtained by Aguirre et al. [47]. Our models indicated that *A*. *cachamai* females emerged with 56 ± 2 mature eggs and that their storage capacity was 58 ± 2 eggs, and that *A*. *lapachosus* females emerged with 57 ± 2 eggs, and their storage capacity was 59 ± 2 eggs. Therefore, both species emerged with their maximum egg storage capacity.

Synovigenic parasitoids possess a variety of adaptations that reduce the risk of egg limitation and extend their lifespan (variable egg production rates, host acceptance or rejection, superparasitism of hosts, adjustable clutch size, egg resorption, and host feeding) [26]. The egg production rate (*g*) of *A*. *cachamai* females decreased with increasing female age while the egg production rate for *A*. *lapachosus* increased with increasing female age. To the best of our knowledge, there are few studies that provide information about how egg production is affected by female age. In addition, most of the available information is for experimental designs where females received an excess of hosts. For example, Manzano et al. [78] reported that the egg production rate of *Cosmocomoidea annulicornis* (Ogloblin) (Hymenoptera: Mymaridae) females is affected by age. The lowest egg load observed is when the females are 1 and 12 days old and the highest when females are 4, 5, and 8 days old [79]. In the pro-ovigenic egg parasitoid *Anagrus virlai* Triapitsyn (Mymaridae), the number of parasitized eggs decreases as the female ages. Strictly pro-ovigenic parasitoids emerge with their complete egg supply and do not develop any additional eggs after emergence. In *A*. *virlai*, most of the eggs were laid by females between days 1–5; however, some females also exhibited a second egg maturation process starting on day 3 of their lives. This suggests that there is some level of flexibility in their reproductive behavior, allowing them to produce additional eggs after emergence [34]. Palottini [62], using a similar experimental design and statistical analysis to the one we employed, found that the egg production rate of *Cosmocomoidea* [as *Gonatocerus*] sp. “clado 1” (Mymaridae) aff. *C*. *tuberculifemur* (Ogloblin) is 0.78 days^-1^, meaning that the egg production rate decreases with female age. We also determined that both species needed time to replenish their egg supply when the oviposition rate was high (S1–S8 Figs). *Anagyrus lapachosus* females had a lower egg resorption threshold (*u*) than *A*. *cachamai* females but shared the same egg storage capacity (*C*). Egg resorption by parasitoids may be a mechanism to remove unviable eggs [80] or to recycle nutrients [81]. Most likely, *A*. *cachamai* and *A*. *lapachosus* females experienced egg resorption when host densities were too low or when the hosts were unsuitable and could not support parasitoid development.

Our data provided evidence that the risk of egg limitation was higher for *A*. *cachamai* females than *A*. *lapachosus*, as egg maturation decreased with increasing age in *A*. *cachamai* females. *Anagyrus lapachosus* females exhibited two biological traits that granted them “flexibility” over *A*. *cachamai* females during the oviposition process: 1) an increasing egg production rate (*g*) as the female’s age increased, and 2) females gained experience throughout their lives through interaction with the host.

Functional response experiments are usually carried out for a short period of time (1–48 hours), ignoring that the wasp presents non-foraging behaviors until she is ready to begin host foraging (e.g. maturing or resorbing eggs, resting, grooming, exploring the experimental arena, etc.) [6]. The problem of non-foraging behaviors during functional response experiments can be addressed with the explicit inclusion of non-foraging mechanisms into the functional response models. Likewise, experimental trials should be long enough to allow for the expression of egg production, resting, and other typical non-foraging behaviors. This approach can effectively tackle the issue stemming from the occurrence of non-foraging behavior in parasitoids during functional response experiments. However, it could lead to intricate models that pose challenges regarding data fitting and interpretation. In this work, thanks to the use of Markovian models combined with Bayesian statistics, it was possible to deal with non-foraging behavior when measuring a parasitoid’s functional response.

## Conclusions

The presented methodology has broad application and the potential to increase understanding of the reproductive biology of a wide variety of parasitoid species. From an applied perspective, our developed models have implications for the use of parasitoids as biological control agents. Unlike classical functional response methodology, we assessed candidate species according to their physiology and prior experiences. Using this methodological approach to predict the success of parasitoids as control agents will increase the amount of information obtained from the studied potential biological control species leading to more effective and safe agent selection.

The most significant result concerning the biological control agents *A*. *cachamai* and *A*. *lapachous* was that they showed learning capacity in consuming their host, *Hypogeococcus* sp., which was reflected in variants of the type III functional response they exhibited. However, in the parasitoid *A*. *lapachosus*, this learning was cumulative, suggesting long-term memory. In *A*. *cachamai*, after day two, the parasitoids were limited by their availability of eggs, making the functional response secondary, while in the case of *A*. *lapachosus*, after day two, the number of hosts attacked depended on the egg stock and the females’ previous experience.

## Supporting information

S1 Fig Observed functional response of *Anagyrus cachamai* parasitizing nymphs of *Hypogeococcus* sp. (A-K) Solid line indicates the mean estimation of functional response for model *C*5 at different ages of female lifespan (1–11 days), grey line indicates its credibility interval, and dashed line indicates the *a posteriori* credibility interval for individual measurements. Dark circles are the observed number of emerged parasitoids; (L) estimated functional response for model *C*5 from day 1 to11.
(TIF)

S2 Fig Observed functional response of *Anagyrus cachamai* parasitizing nymphs of *Hypogeococcus* sp. (A-K) Solid line indicates the mean estimation of functional response for model *C*7 at different ages of female lifespan (1–11 days), grey line indicates its credibility interval, and dashed line indicates the *a posteriori* credibility interval for individual measurements. Dark circles are the observed number of emerged parasitoids; (L) estimated functional response for model *C*7 from day 1 to11.
(TIF)

S3 Fig Observed functional response of *Anagyrus cachamai* parasitizing nymphs of *Hypogeococcus* sp. (A-K) Solid line indicates the mean estimation of functional response for model *E*5 at different ages of female lifespan (1–11 days), grey line indicates its credibility interval, and dashed line indicates the *a posteriori* credibility interval for individual measurements. Dark circles are the observed number of emerged parasitoids; (L) estimated functional response for model *E*5 from day 1 to11.
(TIF)

S4 Fig Observed functional response of *Anagyrus cachamai* parasitizing nymphs of *Hypogeococcus* sp. (A-K) Solid line indicates the mean estimation of functional response for model *E*7 at different ages of female lifespan (1–11 days), grey line indicates its credibility interval, and dashed line indicates the *a posteriori* credibility interval for individual measurements. Dark circles are the observed number of emerged parasitoids; (L) estimated functional response for model *E*7 from day 1 to11.
(TIF)

S5 Fig Observed functional response of *Anagyrus lapachosus* parasitizing nymphs of *Hypogeococcus* sp. (A-L) Solid line indicates the mean estimation of functional response for model *D*4 at different ages of female lifespan (1–12 days), grey line indicates its credibility interval, and dashed line indicates the *a posteriori* credibility interval for individual measurements. Dark circles are the observed number of emerged parasitoids; (M) estimated functional response for model *D*4 from day 1 to12.
(TIF)

S6 Fig Observed functional response of *Anagyrus lapachosus* parasitizing nymphs of *Hypogeococcus* sp. (A-L) Solid line indicates the mean estimation of functional response for model *D*5 at different ages of female lifespan (1–12 days), grey line indicates its credibility interval, and dashed line indicates the *a posteriori* credibility interval for individual measurements. Dark circles are the observed number of emerged parasitoids; (M) estimated functional response for model *D*5 from day 1 to12.
(TIF)

S7 Fig Observed functional response of *Anagyrus lapachosus* parasitizing nymphs of *Hypogeococcus* sp. (A-L) Solid line indicates the mean estimation of functional response for model *D*6 at different ages of female lifespan (1–12 days), grey line indicates its credibility interval, and dashed line indicates the *a posteriori* credibility interval for individual measurements. Dark circles are the observed number of emerged parasitoids; (M) estimated functional response for model *D*6 from day 1 to12.
(TIF)

S8 Fig Observed functional response of *Anagyrus lapachosus* parasitizing nymphs of *Hypogeococcus* sp. (A-L) Solid line indicates the mean estimation of functional response for model *D*7 at different ages of female lifespan (1–12 days), grey line indicates its credibility interval, and dashed line indicates the *a posteriori* credibility interval for individual measurements. Dark circles are the observed number of emerged parasitoids; (M) estimated functional response for model *D*7 from day 1 to12.
(TIF)

S1 Table Deviance information criterion (DIC) of the 48 tested models. (DOCX)

S2 Table Parameters of the selected models for the parasitoid species *Anagyrus cachamai*. (DOCX)

S3 Table Parameters of the selected models for the parasitoid species *Anagyrus lapachosus*. (DOCX)

S1 File Appendix. (DOCX)

## References

[1] Holling CS. Some characteristics of simple types of predation and parasitism. Can Entomol. 1959;91: 385–398. doi: 10.4039/Ent91385-7

[2] Solomon ME. The natural control of animal populations. J Anim Ecol. 1949;18: 1–35. doi: 10.2307/1578

[3] Oaten A, Murdoch WW. Functional response and stability in predator-prey systems. Am Nat. 1975;109: 289–298. Available: http://www.jstor.org/stable/2459695

[4] Uiterwaal SF, Lagerstrom IT, Lyon SR, DeLong JP. FoRAGE database: A compilation of functional responses for consumers and parasitoids. Ecology. 2022;103: e3706. doi: 10.1002/ecy.3706 35362561

[5] Rosenbaum B, Rall BC. Fitting functional responses: Direct parameter estimation by simulating differential equations. Methods Ecol Evol. 2018;9: 2076–2090. doi: 10.1111/2041-210X.13039

[6] Griffen BD. Considerations when applying the consumer functional response measured under artificial conditions. Front Ecol Evol. 2021. p. 461. Available: https://www.frontiersin.org/article/10.3389/fevo.2021.713147

[7] Rogers D. Random search and insect population models. J Anim Ecol. 1972;41: 369–383. doi: 10.2307/3474

[8] Real LA. Ecological determinants of functional response. Ecology. 1979;60: 481–485. doi: 10.2307/1936067

[9] Bruzzone OA, Aguirre MB, Hill JG, Virla EG, Logarzo G. Revisiting the influence of learning in predator functional response, how it can lead to shapes different from type III. Ecol Evol. 2022;12: e8593. 10.1002/ece3.8593 35222965 PMC8844134

[10] Jeschke JM, Kopp M, Tollrian R. Predator functional responses: discriminating between handling and digesting prey. Ecol Monogr. 2002;72: 95–112. doi: 10.1890/0012-9615(2002)072[0095:PFRDBH]2.0.CO;2

[11] Casas J, McCauley E. Daily foraging cycles create overlapping time-scales in functional responses. Oikos. 2012;121: 1966–1976. doi: 10.1111/j.1600-0706.2012.20184.x

[12] Takahashi F. Functional response to host density in a parasitic wasp, with reference to population regulation. Res Popul Ecol (Kyoto). 1968;10: 54–68. doi: 10.1007/BF02514734

[13] Collins MD, Ward SA, Dixon AFG. Handling time and the functional response of *Aphelinus thomsoni*, a predator and parasite of the aphid *Drepanosiphum platanoidis*. J Anim Ecol. 1981;50: 479–487. doi: 10.2307/4069

[14] Bezemer TM, Mills NJ. Host density responses of *Mastrus ridibundus*, a parasitoid of the codling moth, *Cydia pomonella*. Biol Control. 2001;22: 169–175. 10.1006/bcon.2001.0963

[15] Chong J-H, Oetting RD. Progeny fitness of the mealybug parasitoid *Anagyrus* sp. nov. nr. Sinope (Hymenoptera: Encyrtidae) as affected by brood size, sex ratio, and host quality. Florida Entomol. 2007;90: 656–664. doi: 10.1653/0015-4040(2007)90[656:PFOTMP]2.0.CO;2

[16] Irvin N, Suarez-Espinoza J, Hoddle M. Functional response of *Gonatocerus ashmeadi* and the “new association” parasitoid *G*. *tuberculifemur* attacking eggs of *Homalodisca vitripennis*. Environ Entomol. 2009;38: 1634–1641. doi: 10.1603/022.038.0616 20021759

[17] Kaçar G, Wang XG, Biondi A, Daane KM. Linear functional response by two pupal *Drosophila* parasitoids foraging within single or multiple patch environments. PLoS One. 2017;12: 1–17. doi: 10.1371/journal.pone.0183525 28829796 PMC5567721

[18] Bellows TS. Effects of host age and host availability on developmental period, adult size, sex ratio, longevity and fecundity in *Lariophagus distinguendus* Förster (Hymenoptera: Pteromalidae). Res Popul Ecol (Kyoto). 1985;27: 55–64.

[19] Eggleston DB. Behavioural mechanisms underlying variable functional responses of blue crabs, *Callinectes sapidus* feeding on juvenile oysters, *Crassostrea virginica*. J Anim Ecol. 1990;59: 615–630. doi: 10.2307/4884

[20] Sahragard A, Jervis MA, Kidd NAC. Influence of host availability on rates of oviposition and host-feeding, and on longevity in *Dicondylus indianus* Olmi (Hym., Dryinidae), a parasitoid of the Rice Brown Planthopper, *Nilaparvata lugens* Stål (Hem., Delphacidae). J Appl Entomol. 1991;112: 153–162. doi: 10.1111/j.1439-0418.1991.tb01041.x

[21] Varone L, Bruzzone O, Logarzo GA. Egg limitation and the functional response of the parasitoid *Campoletis grioti* (Hym: Ichneumonidae). Biocontrol Sci Technol. 2007;17: 945–955. doi: 10.1080/09583150701596289

[22] Wheeler D. The role of nourishment in oogenesis. Annu Rev Entomol. 1996;41: 407–431. doi: 10.1146/annurev.en.41.010196.002203 15012335

[23] Papaj DR. Ovarian dynamics and host use. Annu Rev Entomol. 2000;45: 423–448. doi: 10.1146/annurev.ento.45.1.423 10761584

[24] Krysko D V, Diez-Fraile A, Criel G, Svistunov AA, Vandenabeele P, D’Herde K. Life and death of female gametes during oogenesis and folliculogenesis. Apoptosis. 2008;13: 1065–1087. doi: 10.1007/s10495-008-0238-1 18622770

[25] Vézina F, Salvante KG. Behavioral and physiological flexibility are used by birds to manage energy and support investment in the early stages of reproduction. Curr Zool. 2010;56: 767–792. doi: 10.1093/czoolo/56.6.767

[26] Phillips CB, Kean JM. Response of parasitoid egg load to host dynamics and implications for egg load evolution. J Evol Biol. 2017;30: 1313–1324. 10.1111/jeb.13095 28425140

[27] Jervis MA, Heimpel GE, Ferns PN, Harvey JA, Kidd NAC. Life‐history strategies in parasitoid wasps: a comparative analysis of ‘ovigeny’. J Anim Ecol. 2001;70: 442–458. doi: 10.1046/j.1365-2656.2001.00507.x

[28] Godfray HCJ, Godfray HCJ. Parasitoids: Behavioral and evolutionary ecology. Princeton University Press; 1994.

[29] Richard R, Casas J. A quantitative framework for ovarian dynamics. Funct Ecol. 2012;26: 1399–1408. doi: 10.1111/j.1365-2435.2012.02050.x

[30] XU L, ZHOU C, XIAO Y, ZHANG P, TANG Y, Xu Y. Insect oviposition plasticity in response to host availability: the case of the tephritid fruit fly *Bactrocera dorsalis*. Ecol Entomol. 2012;37: 446–452. doi: 10.1111/j.1365-2311.2012.01383.x

[31] Rosenheim JA. An evolutionary argument for egg limitation. Evolution. 1996;50: 2089–2094. doi: 10.1111/j.1558-5646.1996.tb03595.x 28565601

[32] Rosenheim JA, Jepsen SJ, Matthews CE, Smith DS, Rosenheimk MR. Time limitation, egg limitation, the cost of oviposition, and lifetime reproduction by an insect in nature. Am Nat. 2008;172: 486–496. doi: 10.1086/591677 18793093

[33] Wäckers FL, van Rijn PCJ, Heimpel GE. Honeydew as a food source for natural enemies: Making the best of a bad meal? Biol Control. 2008;45: 176–184. 10.1016/j.biocontrol.2008.01.007

[34] Hill JG, Aguirre MB, Bruzzone OA, Virla EG, Luft Albarracin E. Influence of adult diet on fitness and reproductive traits of the egg parasitoid *Anagrus virlai* (Hymenoptera: Mymaridae), a potential biocontrol agent against the corn leafhopper. J Appl Entomol. 2020;n/a. doi: 10.1111/jen.12762

[35] Jervis MA, Copland MJW. The life cycle. Insect Nat enemies Pract approaches to their study Eval. 1996; 63–161.

[36] Bernstein C, Jervis M. Food-searching in parasitoids: The dilemma of choosing between ‘immediate’ or future fitness gains. Behavioral ecology of insect parasitoids. 2008. pp. 129–171. doi: 10.1002/9780470696200.ch7

[37] Jervis M, Ellers J, Harvey J. Resource Acquisition, allocation, and utilization in parasitoid reproductive strategies. Annu Rev Entomol. 2008;53: 361–385. doi: 10.1146/annurev.ento.53.103106.093433 17877453

[38] Lotka AJ. Elements of physical biology. Sci Prog Twent Century. 1926;21: 341–343. Available: http://www.jstor.org/stable/43430362

[39] Nicholson AJ, Bailey VA. The balance of animal populations. Part I. Proc Zool Soc London. 1935;105: 551–598. doi: 10.1111/j.1096-3642.1935.tb01680.x

[40] Hassell MP. The dynamics of arthropod predator-prey systems. Monogr Popul Biol. 1978; III–VII, 1–237. Available: http://europepmc.org/abstract/MED/732858 732858

[41] Hassell MP. The spatial and temporal dynamics of host-parasitoid interactions. New York: Oxford University Press; 2000.

[42] Murdoch WW, Briggs CJ, Nisbet RM. Consumer-resource dynamics. Princeton University Press; 2003.

[43] Schreiber SJ, Vejdani M. Handling time promotes the coevolution of aggregation in predator-prey systems. Proceedings Biol Sci. 2006;273: 185–191. doi: 10.1098/rspb.2005.3236 16555786 PMC1560034

[44] Kon R, Sebastian S. Multiparasitoid-host interactions with egg-limited encounter rates. SIAM J Appl Math. 2009;69: 959–976. doi: 10.1137/080717006

[45] Rosenheim JA. Stochasticity in reproductive opportunity and the evolution of egg limitation in insects. Evolution. 2011;65: 2300–2312. doi: 10.1111/j.1558-5646.2011.01305.x 21790576

[46] Triapitsyn S V, Aguirre MB, Logarzo GA, Hight SD, Ciomperlik MA, Rugman-Jones PF, et al. Complex of primary and secondary parasitoids (Hymenoptera: Encyrtidae and Signiphoridae) of *Hypogeococcus* spp. mealybugs (Hemiptera: Pseudococcidae) in the New World. Florida Entomol. 2018;101: 411–434.

[47] Aguirre MB, Logarzo GA, Triapitsyn S V, Diaz-Soltero H, Hight SD, Bruzzone OA. Analysis of biological traits of *Anagyrus cachamai* and *Anagyrus lapachosus* to assess their potential as biological control candidate agents against Harrisia cactus mealybug pest in Puerto Rico. BioControl. 2019;64: 539–551. doi: 10.1007/s10526-019-09956-y

[48] Poveda-Martínez D, Aguirre MB, Logarzo G, Hight SD, Triapitsyn S, Diaz-Sotero H, et al. Species complex diversification by host plant use in an herbivorous insect: The source of Puerto Rican cactus mealybug pest and implications for biological control. Ecol Evol. 2020;10: 10463–10480. 10.1002/ece3.6702 33072273 PMC7548167

[49] Zimmermann HG, Pérez M, Cuen S, Mandujano M, Golubov J. The South American mealybug that threatens North American cacti. Cactus Succul J. 2010;82: 105–107. doi: 10.2985/015.082.0303

[50] Zimmermann HG, Pérez S, Cuen M. La amenaza de los piojos harinosos *Hypogeococcus pungens* e *Hypogeococcus festerianus* (Hemiptera: Pseudococcidae) a las cactáceas mexicanas y del Caribe. Cact y Suculentas Mex. 2010;55: 4–17.

[51] Segarra-Carmona AE, Ramírez-Lluch A, Cabrera-Asencio I, Jiménez-López AN. First report of a new invasive mealybug, the Harrisia cactus mealybug *Hypogeococcus pungens* (Hemiptera: Pseudococcidae). J Agric Univ Puerto Rico. 2010;94: 183–187.

[52] Poveda-Martínez D, Salinas NA, Aguirre MB, Sánchez-Restrepo AF, Hight S, Diaz-Soltero H, et al. Genomic and ecological evidence shed light on the recent demographic history of two related invasive insects. Sci Rep. 2022;12: 19629. doi: 10.1038/s41598-022-21548-y 36385480 PMC9669014

[53] Kalinkat G, Schneider FD, Digel C, Guill C, Rall BC, Brose U. Body masses, functional responses and predator–prey stability. Ecol Lett. 2013;16: 1126–1134. doi: 10.1111/ele.12147 23819684

[54] Poveda-Martínez D, Aguirre MB, Logarzo G, Calderón L, de la Colina A, Hight S, et al. Untangling the *Hypogeococcus pungens* species complex (Hemiptera: Pseudococcidae) for Argentina, Australia, and Puerto Rico based on host plant associations and genetic evidence. PLoS One. 2019;14: e0220366. Available: 10.1371/journal.pone.0220366 31344099 PMC6657911

[55] Paris Q. The Return of von Liebig’s “Law of the Minimum”. Agron J. 1992;84: 1040–1046.

[56] Bruzzone OA, Logarzo GA, Aguirre MB, Virla EG. Intra-host interspecific larval parasitoid competition solved using modelling and bayesian statistics. Ecol Modell. 2018;385: 114–123.

[57] Aguirre MB, Bruzzone OA, Triapitsyn S V, Diaz-Soltero H, Hight SD, Logarzo GA. Influence of competition and intraguild predation between two candidate biocontrol parasitoids on their potential impact against Harrisia cactus mealybug, *Hypogeococcus* sp. (Hemiptera: Pseudococcidae). Sci Rep. 2021;11: 13377. doi: 10.1038/s41598-021-92565-6 34183698 PMC8239034

[58] Real LA. The kinetics of functional response. Am Nat. 1977;111: 289–300.

[59] Gelman A, Carlin JB, Stern HS, Rubin DB. Bayesian data analysis. Texts Stat Sci. 2003;2nd editio: 661.

[60] Gill J. Bayesian methods: A social and behavioral sciences approach. CRC press; 2014.

[61] Fernández-Arhex V, Corley JC. La respuesta funcional: Una revisión y guía experimental. Ecol Austral. 2004;14: 83–93.

[62] Johnson JB, Omland KS. Model selection in ecology and evolution. Trends Ecol Evol. 2004;19: 101–108. doi: 10.1016/j.tree.2003.10.013 16701236

[63] Spiegelhalter DJ, Best NG, Carlin BP, Van Der Linde A. Bayesian measures of model complexity and fit. J R Stat Soc Ser B (Statistical Methodology). 2002;64: 583–639. 10.1111/1467-9868.00353

[64] Gelman A, Carlin JB, Stern HS, Rubin DB. Bayesian data analysis. Chapman and Hall. CRC Texts Stat Sci. 2004.

[65] Burnham KP, Anderson DR. Multimodel inference: Understanding AIC and BIC in model selection. Sociol Methods Res. 2004;33: 261–304. doi: 10.1177/0049124104268644

[66] Cox D, Snell EJ. The analysis of binary data. London: Chapman and Hall. 1989.

[67] Magee L. R 2 measures based on Wald and likelihood ratio joint significance tests. Am Stat. 1990;44: 250–253.

[68] Bruzzone O. GitHub—okktawio/Parasitoids-Egg-Models: Hidden Markov Chain modelization of functional response and egg maduration in parasitoid insects. 2022 [cited 18 Feb 2022]. Available: https://github.com/okktawio/Parasitoids-Egg-Models#readme

[69] Fonnesbeck C, Patil A, Huard D, Salvatier J. PyMC: Bayesian stochastic modelling in python. Astrophysics Source Code Library. 2015. p. ascl:1506.005. Available: https://ui.adsabs.harvard.edu/abs/2015ascl.soft06005F

[70] Vinson SB. The general host selection behavior of parasitoid Hymenoptera and a comparison of initial strategies utilized by larvaphagous and oophagous species. Biol Control. 1998;11: 79–96.

[71] Wajnberg E. Analysis of variations of handling-time in *Trichogramma maidis*. Entomophaga. 1989;34: 397–407. doi: 10.1007/BF02372479

[72] Louâpre P, van Alphen JJM, Pierre J-S. Humans and insects decide in similar ways. PLoS One. 2010;5: e14251. Available: 10.1371/journal.pone.0014251 21170378 PMC2999526

[73] Holling CS. The functional response of predators to prey density and its role in mimicry and population regulation. Mem Entomol Soc Canada. 2012/05/31. 1965;97: 5–60. doi: 10.4039/entm9745fv

[74] Haverkamp A, Smid HM. A neuronal arms race: the role of learning in parasitoid-host interactions. Curr Opin Insect Sci. 2020. 10.1016/j.cois.2020.09.003 32947014

[75] Giunti G, Canale A, Messing RH, Donati E, Stefanini C, Michaud JP, et al. Parasitoid learning: Current knowledge and implications for biological control. Biol Control. 2015;90: 208–219. 10.1016/j.biocontrol.2015.06.007

[76] Thiel A, Schlake S, Kosior D. Omnia tempus habent: Habitat-specific differences in olfactory learning and decision making in parasitic wasps. Anim Cogn. 2013;16: 223–232. 23065185 10.1007/s10071-012-0567-x

[77] Heimpel GE, Neuhauser C, Hoogendoorn M. Effects of parasitoid fecundity and host resistance on indirect interactions among hosts sharing a parasitoid. Ecol Lett. 2003;6: 556–566. 10.1046/j.1461-0248.2003.00466.x

[78] Manzano C, Virla E, Aráoz M, Albarracin E. Ovigeny strategy of the parasitic wasp *Cosmocomoidea annulicornis* (Hymenoptera: Mymaridae): effect of female age, feeding and host availability on reproductive traits. Bull Entomol Res. 2021. doi: 10.1017/S0007485321000766 34486968

[79] Manzano C, Benzal G, Logarzo GA, Coll Araoz M V, Virla EG, Luft Albarracin E. Biological traits of *Cosmocomoidea annulicornis* (Hymenoptera: Mymaridae), an egg parasitoid of the sharpshooter *Tapajosa rubromarginata* (Hemiptera: Cicadellidae), a vector of *Xylella fastidiosa* in citrus orchards. Biol Control. 2021;157: 104589. doi: 10.1016/j.biocontrol.2021.104589

[80] Asplen MK, Byrne DN. Quantification and ultrastructure of oosorption in *Eretmocerus eremicus* (Hymenoptera: Aphelinidae). J Morphol. 2006;267: 1066–1074. 16752404 10.1002/jmor.10459

[81] Rosenheim JA, Heimpel GE, Mangel M. Egg maturation, egg resorption and the costliness of transient egg limitation in insects. Proceedings Biol Sci. 2000;267: 1565–1573. doi: 10.1098/rspb.2000.1179 11007333 PMC1690703

