# Supplementary figures and images for: Effect of egg production dynamics on the functional response of two parasitoids
Source: PLoS One. 2024 Mar 8;19(3):e0283916. doi: 10.1371/journal.pone.0283916 (PMC10923418; doi:10.1371/journal.pone.0283916)

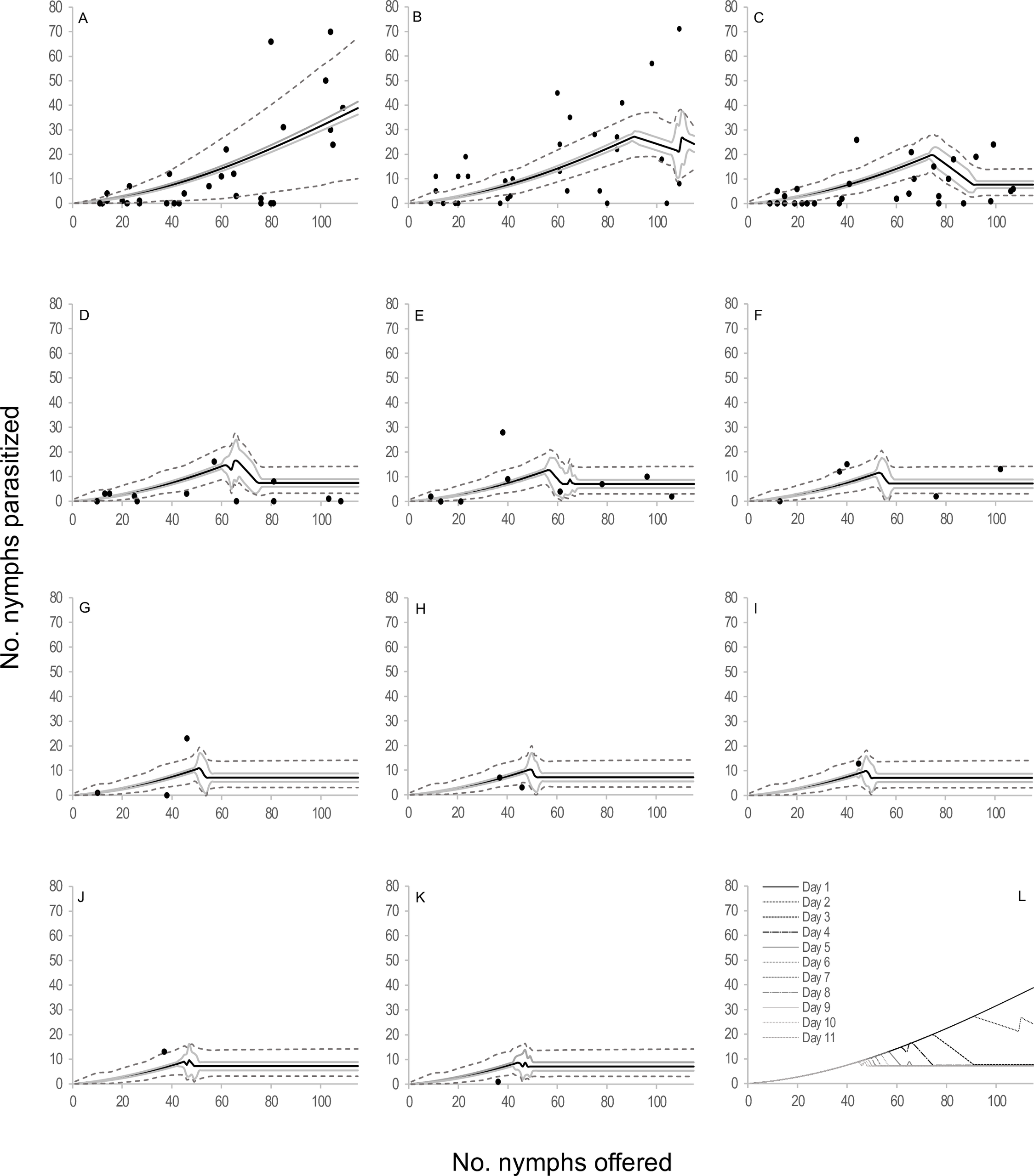

Supplement: S1 Fig — (A-K) Solid line indicates the mean estimation of functional response for model C5 at different ages of female lifespan (1–11 days), grey line indicates its credibility interval, and dashed line indicates the a posteriori credibility interval for individual measurements. Dark circles are the observed number of emerged parasitoids; (L) estimated functional response for model C5 from day 1 to11. (TIF) [file pone.0283916.s001.tif]

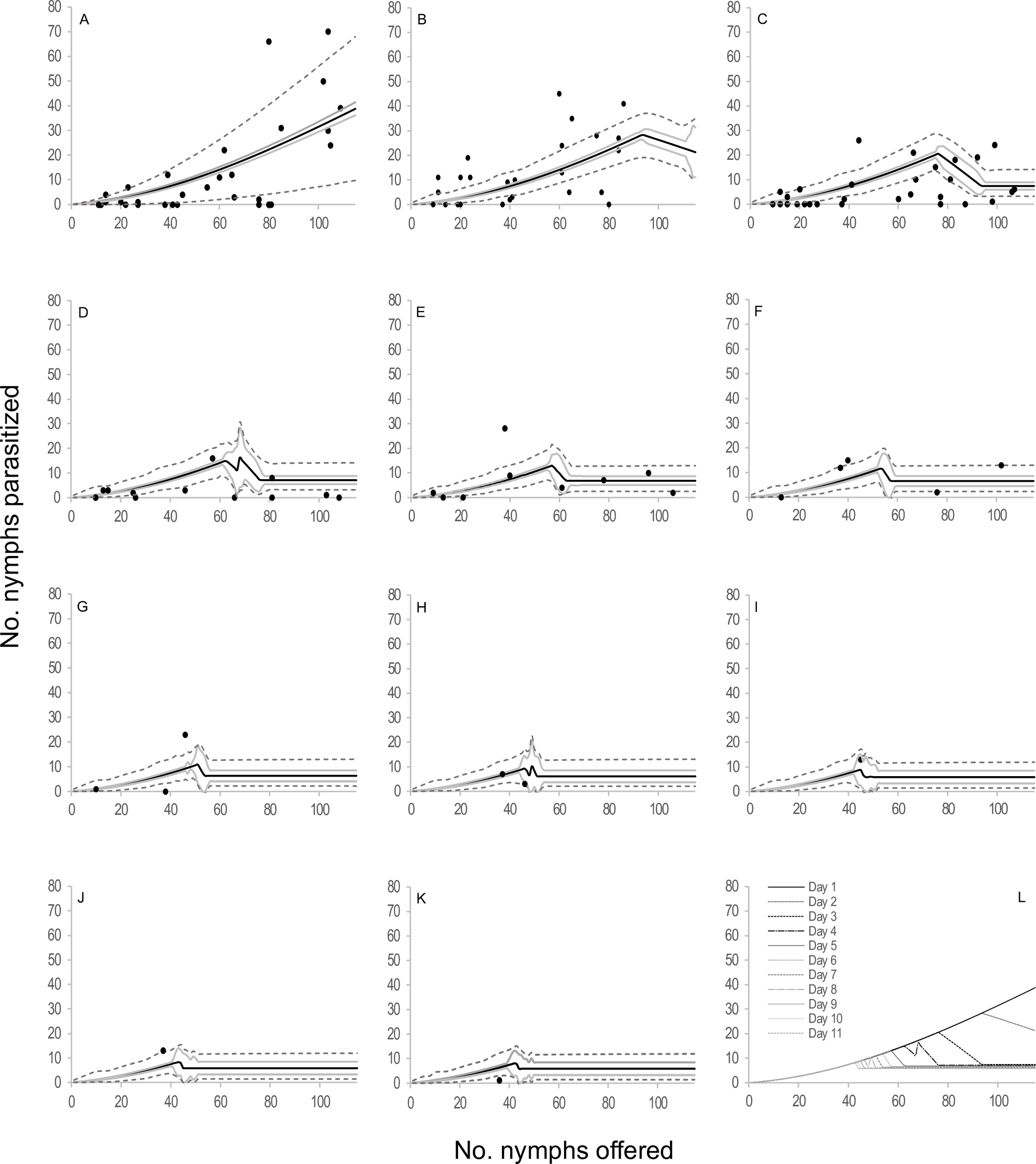

Supplement: S2 Fig — (A-K) Solid line indicates the mean estimation of functional response for model C7 at different ages of female lifespan (1–11 days), grey line indicates its credibility interval, and dashed line indicates the a posteriori credibility interval for individual measurements. Dark circles are the observed number of emerged parasitoids; (L) estimated functional response for model C7 from day 1 to11. (TIF) [file pone.0283916.s002.tif]

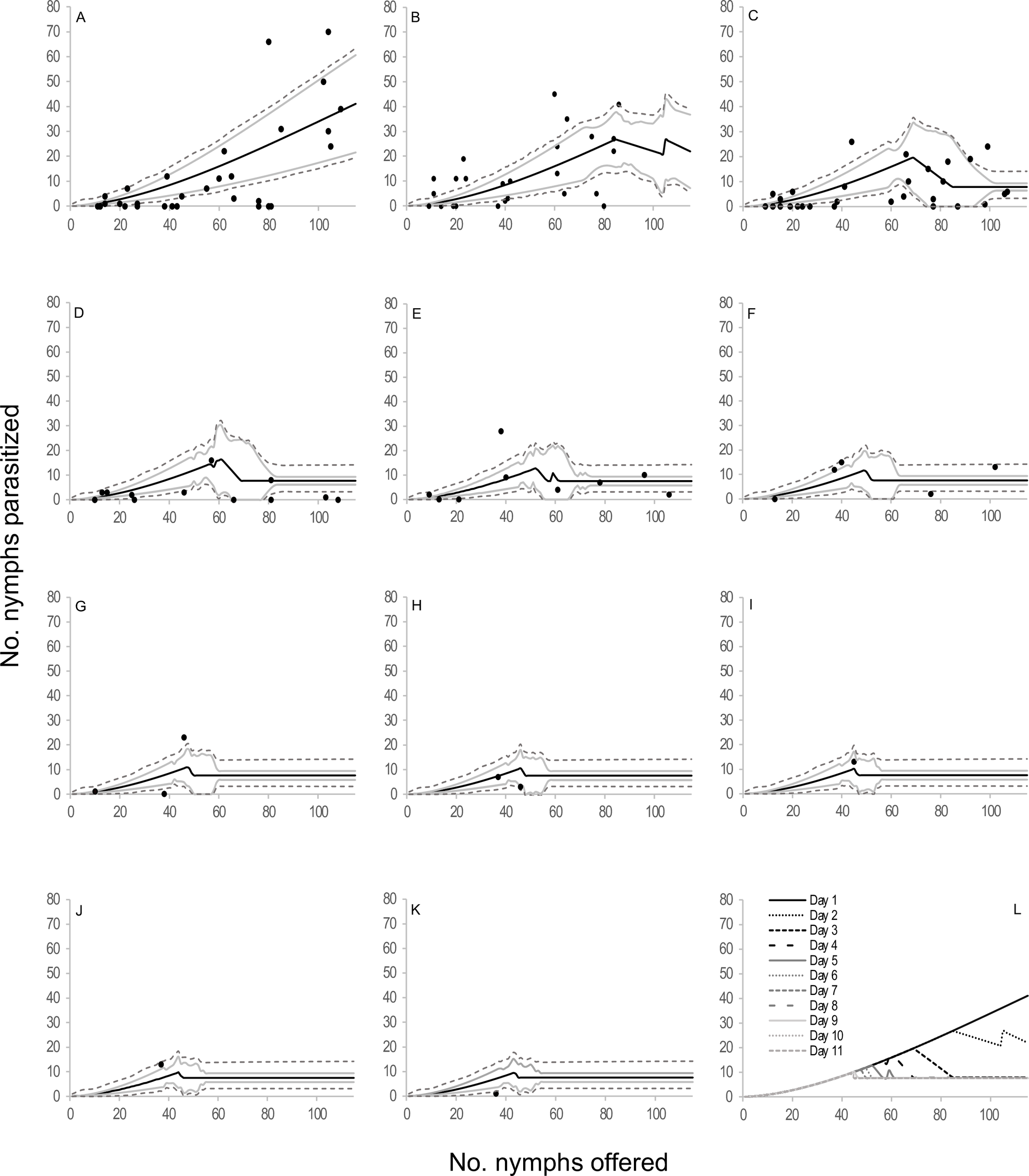

Supplement: S3 Fig — (A-K) Solid line indicates the mean estimation of functional response for model E5 at different ages of female lifespan (1–11 days), grey line indicates its credibility interval, and dashed line indicates the a posteriori credibility interval for individual measurements. Dark circles are the observed number of emerged parasitoids; (L) estimated functional response for model E5 from day 1 to11. (TIF) [file pone.0283916.s003.tif]

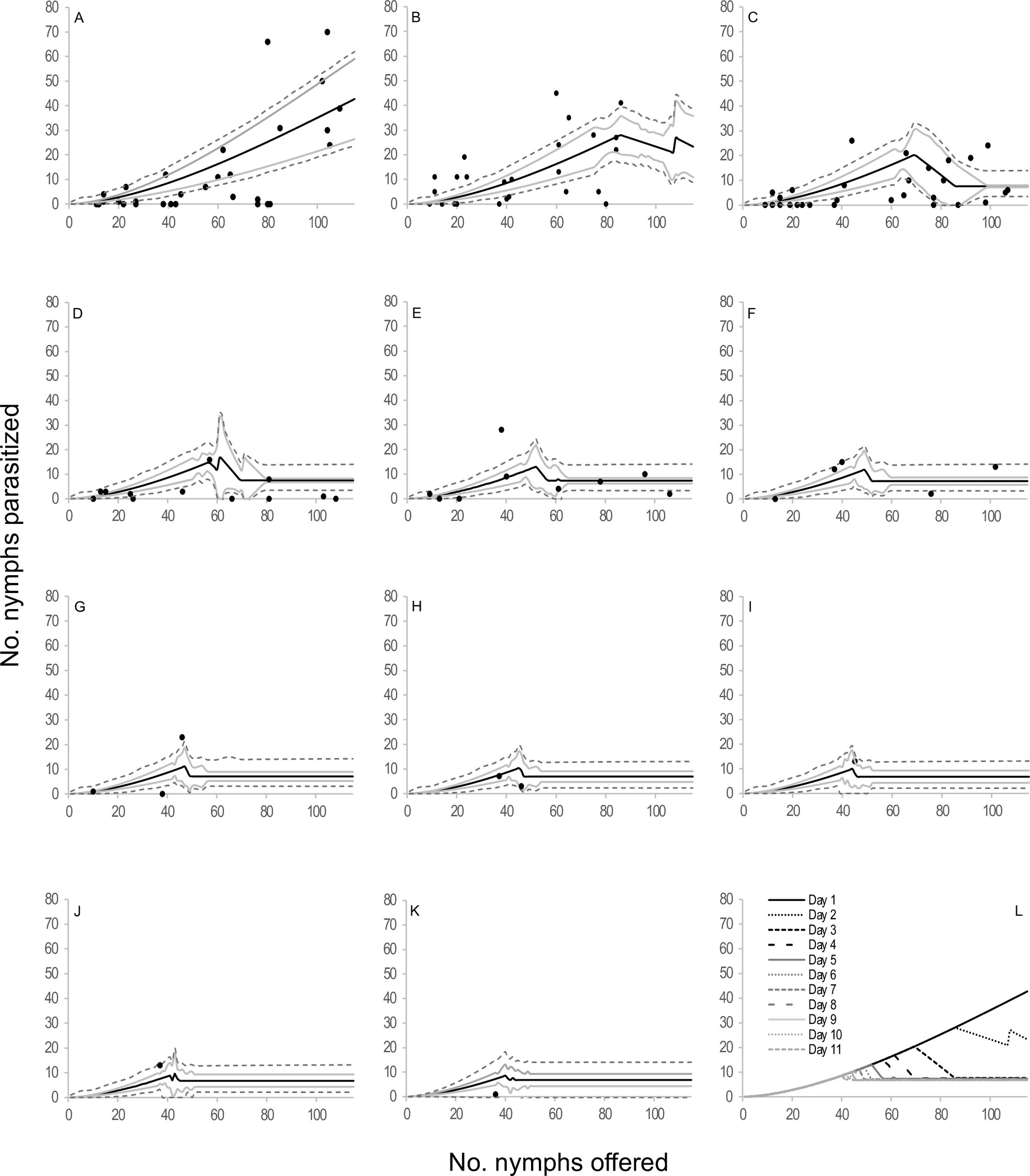

Supplement: S4 Fig — (A-K) Solid line indicates the mean estimation of functional response for model E7 at different ages of female lifespan (1–11 days), grey line indicates its credibility interval, and dashed line indicates the a posteriori credibility interval for individual measurements. Dark circles are the observed number of emerged parasitoids; (L) estimated functional response for model E7 from day 1 to11. (TIF) [file pone.0283916.s004.tif]

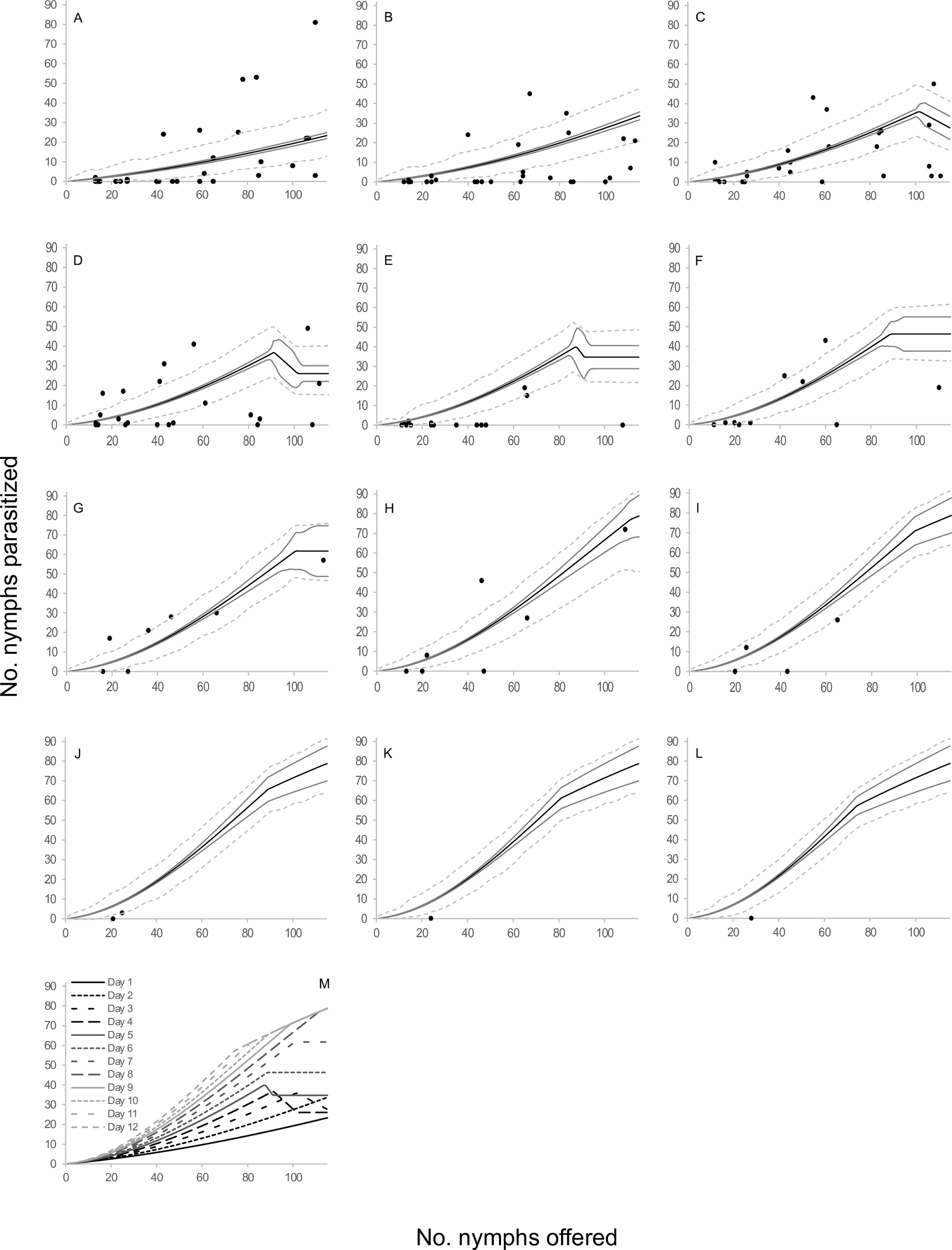

Supplement: S5 Fig — (A-L) Solid line indicates the mean estimation of functional response for model D4 at different ages of female lifespan (1–12 days), grey line indicates its credibility interval, and dashed line indicates the a posteriori credibility interval for individual measurements. Dark circles are the observed number of emerged parasitoids; (M) estimated functional response for model D4 from day 1 to12. (TIF) [file pone.0283916.s005.tif]

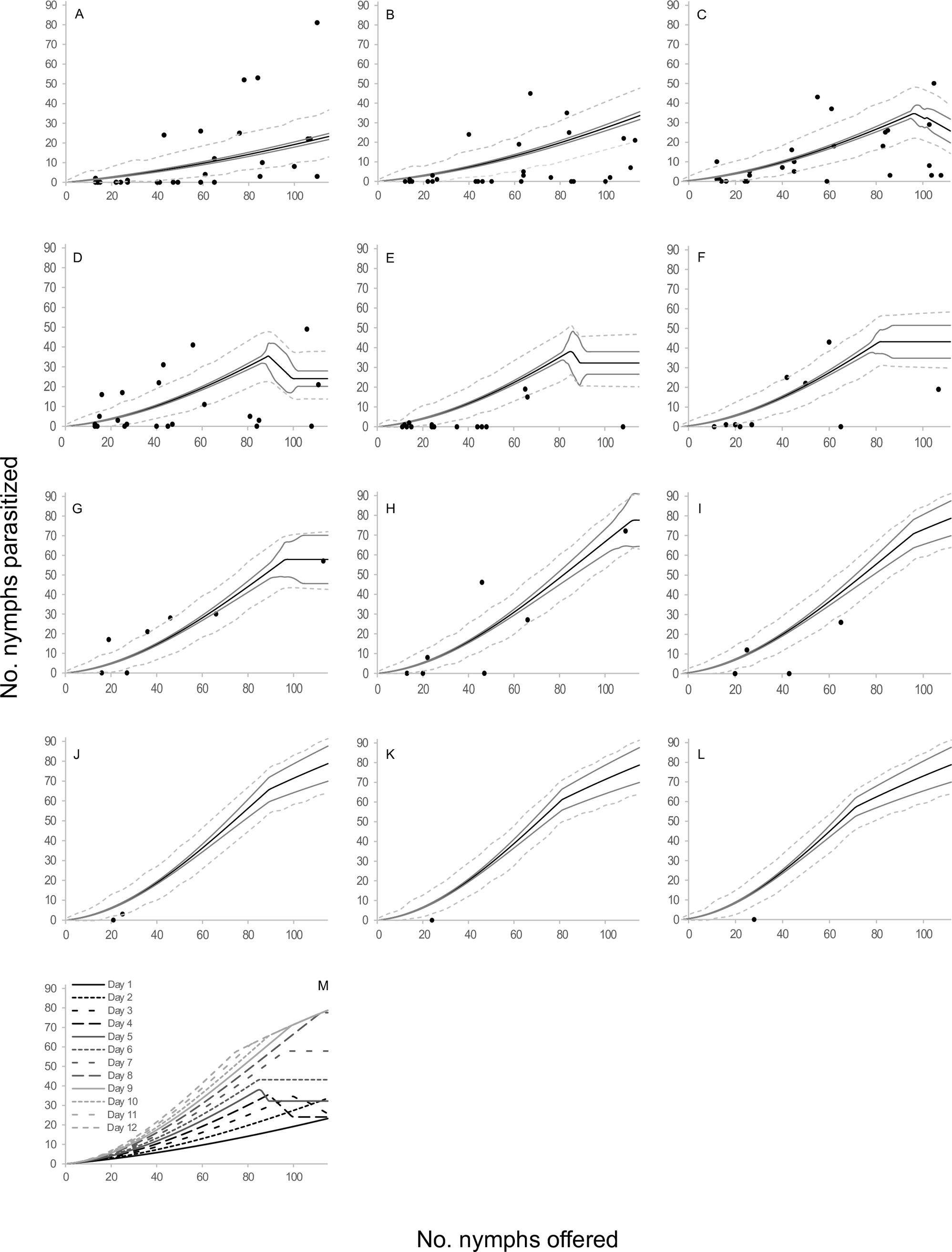

Supplement: S6 Fig — (A-L) Solid line indicates the mean estimation of functional response for model D5 at different ages of female lifespan (1–12 days), grey line indicates its credibility interval, and dashed line indicates the a posteriori credibility interval for individual measurements. Dark circles are the observed number of emerged parasitoids; (M) estimated functional response for model D5 from day 1 to12. (TIF) [file pone.0283916.s006.tif]

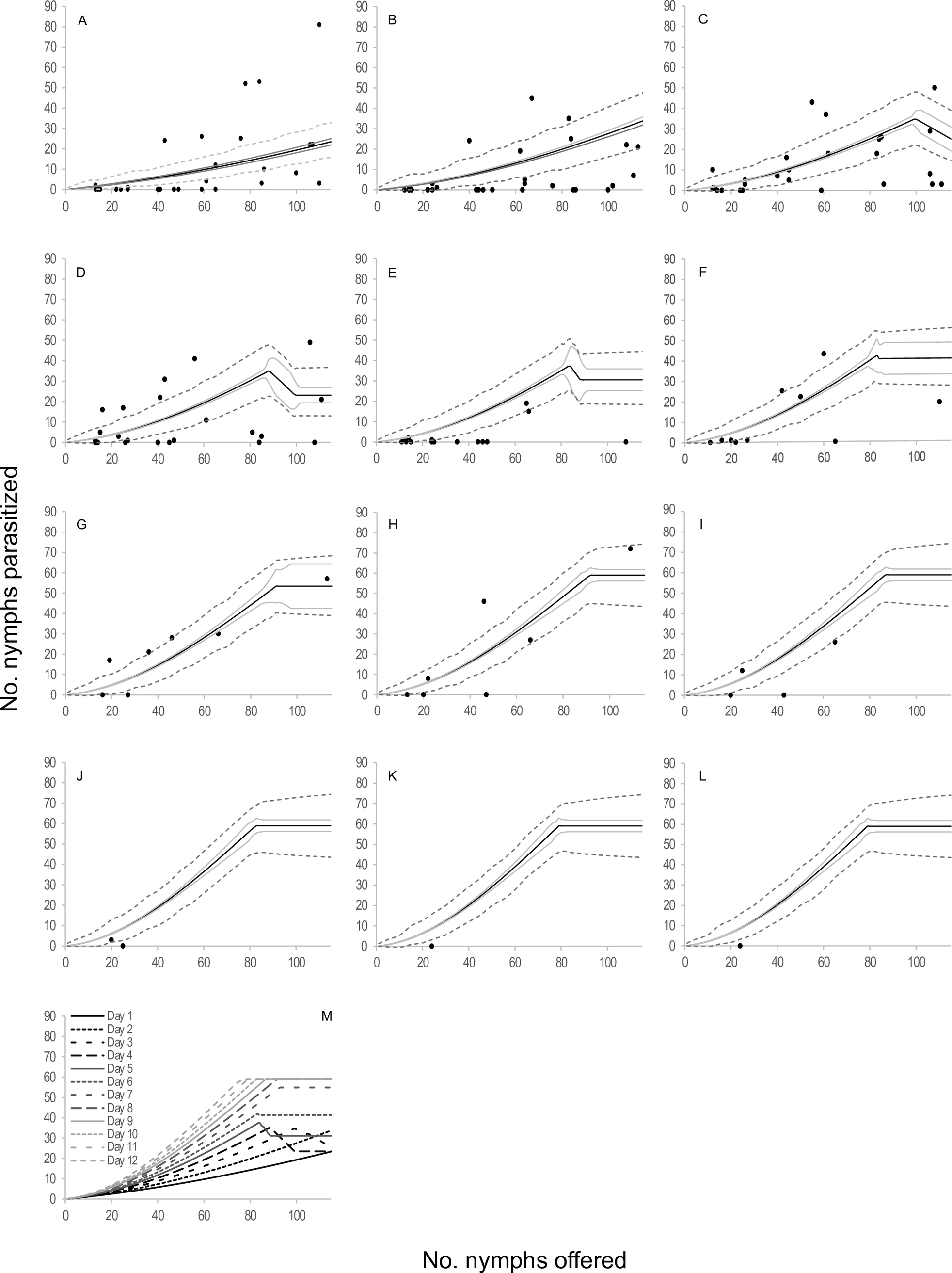

Supplement: S7 Fig — (A-L) Solid line indicates the mean estimation of functional response for model D6 at different ages of female lifespan (1–12 days), grey line indicates its credibility interval, and dashed line indicates the a posteriori credibility interval for individual measurements. Dark circles are the observed number of emerged parasitoids; (M) estimated functional response for model D6 from day 1 to12. (TIF) [file pone.0283916.s007.tif]

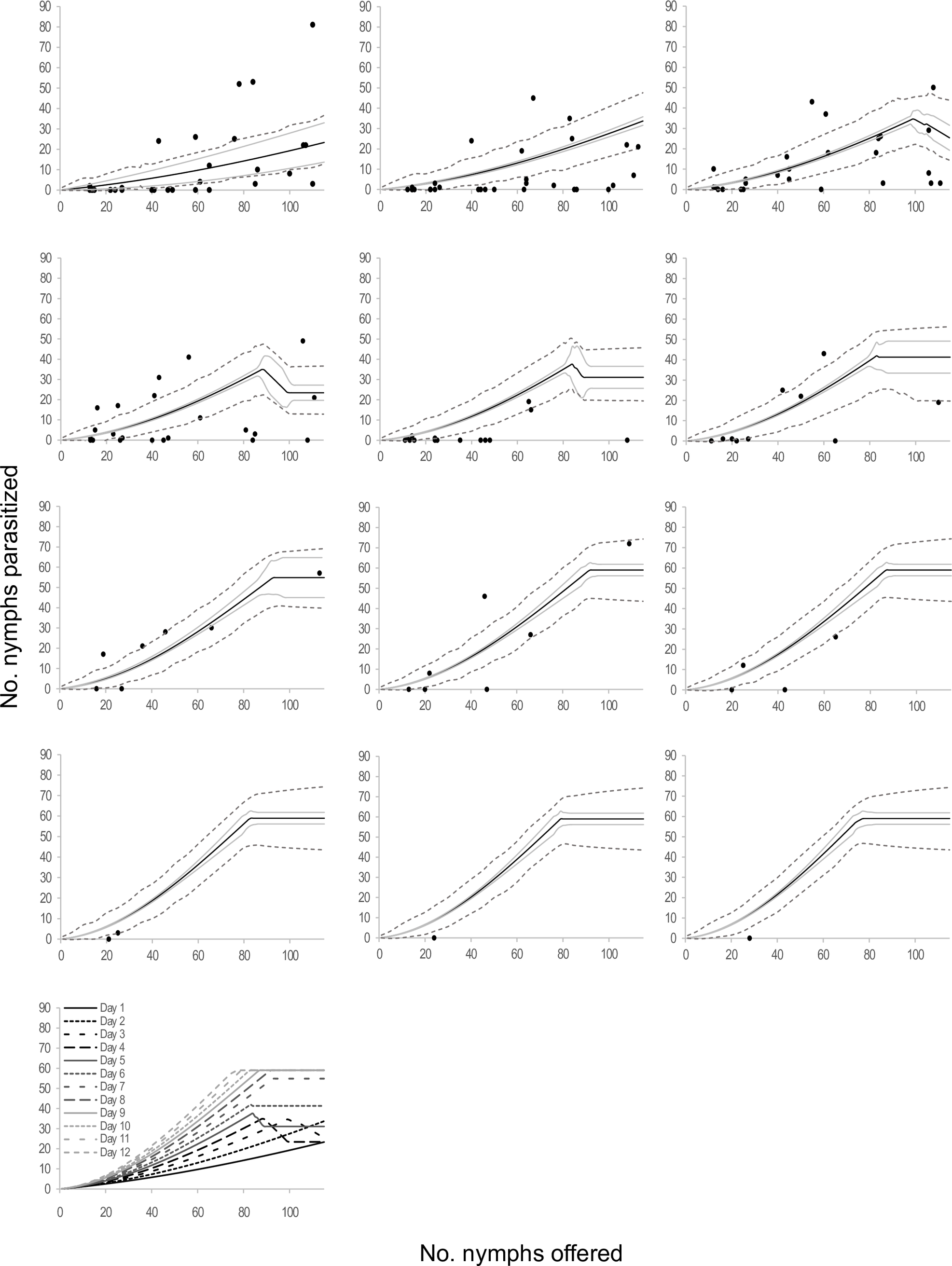

Supplement: S8 Fig — (A-L) Solid line indicates the mean estimation of functional response for model D7 at different ages of female lifespan (1–12 days), grey line indicates its credibility interval, and dashed line indicates the a posteriori credibility interval for individual measurements. Dark circles are the observed number of emerged parasitoids; (M) estimated functional response for model D7 from day 1 to12. (TIF) [file pone.0283916.s008.tif]
