# Supplementary material for: Effect of egg production dynamics on the functional response of two parasitoids
Source: PLoS One. 2024 Mar 8;19(3):e0283916. doi: 10.1371/journal.pone.0283916 (PMC10923418; doi:10.1371/journal.pone.0283916)
Supplement: S1 Table — (DOCX) [file pone.0283916.s009.docx]

**S1 Table. Deviance information criterion (DIC) of the 48 tested models for *Anagyrus cachamai* and *A. lapachosus*.** Asterisks indicate the models that were selected, and the value in parentheses their weight.

| **# Model** | **Egg production**  **module** | **Functional response module** | ***A. cachamai* (DIC)** | ***A. lapachosus* (DIC)** |
| --- | --- | --- | --- | --- |
| 1 | 0 | *A* | 2343.26 | 3265.01 |
| 2 |  | *B* | 2348.76 | 3279.00 |
| 3 |  | *C* | 2340.27 | 3172.32 |
| 4 |  | *D* | 2443.08 | 3136.05 |
| 5 |  | *E* | 2339.00 | 3154.32 |
| 6 |  | *F* | 2528.84 | 3398.80 |
| 7 | 1 | *A* | 2439.28 | 3739.21 |
| 8 |  | *B* | 2098.41 | 3691.44 |
| 9 |  | *C* | 2407.27 | 3564.05 |
| 10 |  | *D* | 2121.61 | 3592.79 |
| 11 |  | *E* | 2435.12 | 3511.53 |
| 12 |  | *F* | 2697.01 | 3684.99 |
| 13 | 2 | *A* | 2138.61 | 3265.00 |
| 14 |  | *B* | 2157.13 | 3279.06 |
| 15 |  | *C* | 2041.57 | 3172.33 |
| 16 |  | *D* | 2094.23 | 3136.20 |
| 17 |  | *E* | 2033.51 | 3153.22 |
| 18 |  | *F* | 2219.89 | 3399.08 |
| 19 | 3 | *A* | 2110.47 | 3241.87 |
| 20 |  | *B* | 2117.05 | 3256.78 |
| 21 |  | *C* | 2039.88 | 3010.09 |
| 22 |  | *D* | 2072.51 | 3046.66 |
| 23 |  | *E* | 2039.53 | 3010.36 |
| 24 |  | *F* | 2485.28 | 3331.99 |
| 25 | 4 | *A* | 2069.07 | 3198.11 |
| 26 |  | *B* | 2087.41 | 3221.72 |
| 27 |  | *C* | 1993.80 | 3007.23 |
| 28 |  | *D* | 2007.95 | **2974.66^*^ (26%)** |
| 29 |  | *E* | 1995.78 | 3005.05 |
| 30 |  | *F* | 2053.26 | 3341.38 |
| 31 | 5 | *A* | 2020.12 | 3198.33 |
| 32 |  | *B* | 2041.32 | 3222.22 |
| 33 |  | *C* | **1933.43^*^ (24%)** | 3007.09 |
| 34 |  | *D* | 1968.37 | **2973.60^*^ (44%)** |
| 35 |  | *E* | **1932.39^*^ (41%)** | 3005.25 |
| 36 |  | *F* | 2028.32 | 3318.07 |
| 37 |  | *A* | 2069.10 | 3198.15 |
| 38 | 6 | *B* | 2087.35 | 3221.94 |
| 39 |  | *C* | 1993.84 | 3007.23 |
| 40 |  | *D* | 2008.03 | **2976.63^*^ (10%)** |
| 41 |  | *E* | 1995.83 | 3005.08 |
| 42 |  | *F* | 2052.84 | 3341.18 |
| 43 |  | *A* | 2020.13 | 3198.20 |
| 44 | 7 | *B* | 2040.99 | 3221.72 |
| 45 |  | *C* | **1933.38^*^ (25%)** | 3007.09 |
| 46 |  | *D* | 1968.28 | **2975.19 (20%)** |
| 47 |  | *E* | **1935.21^*^ (10%)** | 3005.24 |
| 48 |  | *F* | 2031.78 | 3318.48 |
