# Supplementary material for: Effect of egg production dynamics on the functional response of two parasitoids
Source: PLoS One. 2024 Mar 8;19(3):e0283916. doi: 10.1371/journal.pone.0283916 (PMC10923418; doi:10.1371/journal.pone.0283916)
Supplement: S2 Table — (DOCX) [file pone.0283916.s010.docx]

**S2 Table. Parameters of the selected models for the parasitoid species *Anagyrus cachamai*.**

|  |  |  | **Selected models** | | | | | | |
| --- | --- | --- | --- | --- | --- | --- | --- | --- | --- |
|  |  |  | **Model *C*5** |  | **Model *C*7** |  | **Model *E*5** |  | **Model *E*7** |
|  |  |  |  |  |  |  |  |  |  |
| **Functional response module parameters** | |  | FR III  without female experience (attack rate increase linearly with the available hosts number) [1,2] | | |  | FR III without female experience (the attack rate changed with host densities as $an^{s}$ [3]) | | |
|  |  |  |  |  |  |  |  |  |  |
| Attack rate at $n=0 (b)$ | |  | 0.074±0.014 *d*^-1^ |  | 0.073±0.015 *d*^-1^ |  | - |  | - |
| Attack rate change $(a)$ | |  | 0.003±0.000 *d*^-1^ |  | 0.003±0.000 *d*^-1^ |  | - |  | - |
| Attack rate change $(a)$ | |  | - |  | - |  | 0.018±0.005 *d*^-1^ |  | 0.019±0.004 *d*^-1^ |
| Exponent $s (1+q)$ | |  | - |  | - |  | 1.678±0.066 |  | 1.666±0.051 |
| Handling time$(H)$ | |  | 0.005±0.001 *d* |  | 0.005±0.001*d* |  | 0.005±0.001 *d* |  | 0.004±0.001 *d* |
|  |  |  |  |  |  |  |  |  |  |
| **Egg production module parameters** | |  | Sinovigenic females with $g, r$and $u$ |  | Sinovigenic females with $g, r, u$and $C>u$ |  | Sinovigenic females with$g, r$and $u$ |  | Sinovigenic females with $g, r, u$and $C>u$ |
|  |  |  |  |  |  |  |  |  |  |
|  |  |  |  |  |  |  |  |  |  |
| No.  mature eggs after emerge $(e)$ | |  | 55±1 |  | 58±2 |  | 55±2 |  | 58±2 |
| Eggs prod. on the first day $(h_{0})$ | |  | 8±1 |  | 8±1 |  | 8±1 |  | 8±1 |
| Eggs production rate $(g)$ | |  | 0.963±0.036 *d*^-1^ |  | 0.962±0.034 *d*^-1^ |  | 0.982±0.037 *d*^-1^ |  | 0.981±0.036 *d*^-1^ |
| Eggs resorption ratio $(r)$ | |  | 0.310±0.045 |  | 0.311±0.044 |  | 0.334±0.046 |  | 0.332±0.046 |
| Eggs resorption threshold $(u)$ | |  | 19±1 |  | 19±1 |  | 19±1 |  | 19±1 |
| Eggs storage capacity $(C)$ | |  | - |  | 58±2 |  | - |  | 58±2 |

Physical units of the calculated parameters: *d* is days, parameters without units are dimensionless.

**References**

1. Bruzzone OA, Logarzo GA, Aguirre MB, Virla EG. Intra-host interspecific larval parasitoid competition solved using modelling and bayesian statistics. Ecol Modell. 2018;385: 114–123.

2. Aguirre MB, Bruzzone OA, Triapitsyn S V, Diaz-Soltero H, Hight SD, Logarzo GA. Influence of competition and intraguild predation between two candidate biocontrol parasitoids on their potential impact against Harrisia cactus mealybug, *Hypogeococcus* sp. (Hemiptera: Pseudococcidae). Sci Rep. 2021;11: 13377. doi:10.1038/s41598-021-92565-6

3. Kalinkat G, Schneider FD, Digel C, Guill C, Rall BC, Brose U. Body masses, functional responses and predator-prey stability. Ecol Lett. 2013. doi:10.1111/ele.12147
