# Supplementary material for: Effect of egg production dynamics on the functional response of two parasitoids
Source: PLoS One. 2024 Mar 8;19(3):e0283916. doi: 10.1371/journal.pone.0283916 (PMC10923418; doi:10.1371/journal.pone.0283916)
Supplement: S3 Table — (DOCX) [file pone.0283916.s011.docx]

**S3 Table. Parameters of the selected models for the parasitoid species *Anagyrus lapachosus*.**

|  |  |  | **Selected models** | | | | | | |
| --- | --- | --- | --- | --- | --- | --- | --- | --- | --- |
|  |  |  | **Model *D*4** |  | **Model *D*5** |  | **Model *D*6** |  | **Model *D*7** |
|  |  |  |  |  |  |  |  |  |  |
| **Functional response module parameters** | |  | FR III with female experience (attack rate increased linearly with the available host number [1,2]) | | | | | | |
|  |  |  |  |  |  |  |  |  |  |
| Attack rate at $n=0 (b)$ | |  | 0.109±0.010 *d*^-1^ |  | 0.108±0.010 *d*^-1^ |  | 0.110±0.010 *d*^-1^ |  | 0.109±0.011 *d*^-1^ |
| Attack rate change $(a)$ | |  | 0.001±0.000 *d*^-1^ |  | 0.001±0.000 *d*^-1^ |  | 0.001±0.000 *d*^-1^ |  | 0.001±0.000 *d*^-1^ |
| Handling time $(H)$ | |  | 0.004±0.001 *d* |  | 0.004±0.001 *d* |  | 0.004±0.001 *d* |  | 0.004±0.001 *d* |
|  |  |  |  |  |  |  |  |  |  |
| **Egg production module parameters** | |  | Sinovigenic females with $g$, and $r$ |  | Sinovigenic females with $g,r$, and $u$ |  | Sinovigenic females with $g, r, u$, and $C$ |  | Sinovigenic females with $g, r, u,$and $C>u$ |
|  |  |  |  |  |  |  |  |  |  |
|  |  |  |  |  |  |  |  |  |  |
| No. of mature eggs after emerge $(e)$ | |  | 57±2 |  | 57±2 |  | 57±2 |  | 57±2 |
| Eggs prod. on the first day $(h_{0})$ | |  | 11±1 |  | 10±1 |  | 10±1 |  | 10±1 |
| Eggs production rate $(g)$ | |  | 1.333±0.026 *d*^-1^ |  | 1.340±0.025 *d*^-1^ |  | 1.322±0.024 *d*^-1^ |  | 1.328±0.024 *d*^-1^ |
| Eggs resorption ratio $(r)$ | |  | 0.053±0.025 |  | 0.068±0.028 |  | 0.050±0.024 |  | 0.064±0.028 |
| Eggs resorption threshold $(u)$ | |  | - |  | 15±1 |  | - |  | 15±1 |
| Eggs storage capacity $(C)$ | |  | - |  | - |  | 59±2 |  | 59±2 |

Physical units of the calculated parameters: *d* is days, parameters without units are dimensionless.

**References**

1. Bruzzone OA, Logarzo GA, Aguirre MB, Virla EG. Intra-host interspecific larval parasitoid competition solved using modelling and bayesian statistics. Ecol Modell. 2018;385: 114–123.

2. Aguirre MB, Bruzzone OA, Triapitsyn S V, Diaz-Soltero H, Hight SD, Logarzo GA. Influence of competition and intraguild predation between two candidate biocontrol parasitoids on their potential impact against Harrisia cactus mealybug, *Hypogeococcus* sp. (Hemiptera: Pseudococcidae). Sci Rep. 2021;11: 13377. doi:10.1038/s41598-021-92565-6
