# Supplementary material for: Effect of egg production dynamics on the functional response of two parasitoids
Source: PLoS One. 2024 Mar 8;19(3):e0283916. doi: 10.1371/journal.pone.0283916 (PMC10923418; doi:10.1371/journal.pone.0283916)
Supplement: S1 File — (DOCX) [file pone.0283916.s012.docx]

**S1 File. Appendix**

**Data analyses**

Description of models used to estimate the realized lifetime reproductive success of the parasitoids *Anagyrus cachamai* and *A. lapachosus*.

Equation I represents the general model describing the number of eggs laid by a wasp at day $t ( p\left( n,t \right))$ and combines the functional response equation from the functional response module $(f\left( n \right))$, and the egg production equation from the egg production module$(e\left( t \right))$. The symbol $\wedge$ is used to signify the minimum of these two functions, meaning that $p(n,t)$ represents the minimum value between functions $f(n)$ and $e(t)$*.*

$$p(n,t) = f(n) \wedge e(t)$$

(I)

**Functional response module**

This module contained the description of the six proposed functional response equations (*A*-*F*).

- **Equation *A.*** Holling’s type I functional response [1], the number of parasitized hosts is directly proportional to the amount offered:

$f\left( n \right)=ant$

(A)

where $n$ is the number of nymphs offered, $a$ is the attack rate, and *t* is the time the parasitoid is in contact with the host.

- **Equation *B.*** Holling’s type II functional response:

$$f\left( n \right)=\frac{ant}{1+aHn}$$

(B)

$H$ being the handling time.

- **Equation *C.*** The proportion of hosts attacked follows a type III functional response curve with learning in the attack rate $\left( a \right)$ as in Bruzzone et al. [2]. Here, to simplify the parameter estimation, that variable increased linearly with the number of hosts available until it reached one (100% efficiency). Thus, the equation for the functional response module was defined as in Bruzzone et al. [3] and Aguirre et al. [4]:

$$f\left( n \right)=\frac{\left( ant+b \right)n}{1+H\left( an+b \right)n}$$

(C)

which is the Hollings disk equation with a linear function that substitutes the attack rate. Here $a$ (the slope) is the learning rate as a function of the number of nymphs offered, $b$ is the attack rate when female emerges with $n=0$, so the attack rate can increase or decrease as a function of the experience. Always $0<an+b\leq1$. As in the Holling [1] model, here this results in a quadratic relationship between offered hosts and attack rate, but with an additional lineal term which modulates the shape of the functional response curve.

- **Equation *D*.**  Equation *C* does not consider that the female gains experience in the course of her life when interacting with the host, and assumes that the insect’s responsiveness to the stimuli from the host decreases if the time between contacts is very long. In equation *D*, to include the long term female experience in the functional response module, the parameter *n* of the equation *C* now represents the total number of hosts offered during the life of the insect, instead of those offered in each experiment.
- **Equation *E*.** The proportion of hosts attacked by a female follows a generalized functional response curve [5,6], where type II functional response, with a constant attack rate (equation *B*), was modified for an attack rate that changes with host densities as $an^{s}$, leading to a functional response equation that can be written as:

$$f\left( n \right)=\frac{tan^{s}}{1+Han^{s}}$$

(E)

where $s=1+q$*,* $q$ representing the attack component that converts hyperbolic type II functional response $\left( q=0 \right)$ into sigmoid type III functional response $\left( q>0 \right)$.

- **Equation *F.*** As in equation *D*, equation *F* incorporated long term female experience as a result of the interaction with the host by changing the parameter $n$ of equation *E* to represent the total number of hosts offered during the life of the female, instead of the number offered in each experiment.

Egg production module

This module contains the description of the eight proposed egg production equations (0-7).

- **Equation 0 (unlimited egg production).** This equation assumed that the female has an unlimited number of eggs to oviposit, $e\left( t \right)=+\infty$ and $e\left( t \right)\geq f\left( n \right)$, and, as a consequence, the number of laid eggs is the one predicted by the functional response module:

$$p\left( t \right)=f\left( n \right)$$

(0)

- **Equation 1 (strict pro-ovigenic females** [7]**).** The female emerges with a fully developed egg complement, and there is no egg production after emergence. Therefore, the egg production equation proposed was:

$$e\left( t \right)=e\left( t \right)-p\left( n,t \right)$$

(1)

where $e\left( t \right)$ is the number of eggs that a wasp has at the beginning of day $t$and $p\left( n,t \right)$ is the number of eggs laid at day $t$*.* The egg complement can never be negative $\left( e\geq0 \right)$.

Equations 2-7 describe the behavior of synovigenic females [7], which refers to females that emerge with a high proportion of immature eggs or with many eggs that continue maturing throughout their lives.

- **Equation 2.** Female emerges with an egg complement $e\left( t=0 \right)$, and produces $h$ eggs every day. Thus, the egg production module was defined as:

$$e\left( t+1 \right)=e\left( t \right)-p\left( n,t \right)+h$$

(2)

- **Equation 3.** Female emerges with an egg complement $e\left( t=0 \right)$, produces $h\left( t \right)$ eggs every day, and the egg production changes at a steady pace $g$, with$g$ between 0 and infinity. So, the equation describing the eggs production was:

$$e\left( t+1 \right)=e\left( t \right)-p\left( n,t \right)+h\left( t \right)$$

(3)

with $h\left( t \right)=h_{0}g^{t}$, where $h_{0}$ is the egg production at $t=0$, and $g$ the egg production rate, with $g\geq0$. If $g$ takes values between 0 and 1, the egg production rate decreases with time; when $g=1$, the egg production rate remains constant, and if $g>1$, the egg production rate increases with time.

- **Equation 4.** This equation was similar to equation 3, but in this case, the eggs that have not been used are resorbed. Thus, the egg production module was defined as:

$$e\left( t+1 \right)=\left( r \right)\left( e\left( t \right)-p\left( n,t \right) \right)+h\left( t \right)$$

(4)

where $r$ is the proportion of eggs reabsorbed.

- **Equation 5.** As in equation 4, the eggs that have not been used are reabsorbed, but for reabsorption to exist, the number of remaining eggs from one day to another $\left( e\left( t \right)-p\left( n,t \right) \right)$ must exceed a threshold $u$:

$$e\left( t+1 \right)=\delta_{u}(r(e\left( t \right)-p\left( n,t \right))+h(t))+\left( 1-\delta_{u} \right)\left( e\left( t \right)-p\left( n,t \right)+h\left( t \right) \right)$$

$$with \delta_{u}=(e\left( t \right)-p(n,t)>u)$$

(5)

where $\delta_{u}$ is a Kronecker delta that turns 1 when the difference between the egg stock as a function of time $e(t)$ minus the consumed eggs $p(n,t)$ is higher than the threshold $u$, and is zero otherwise. Thus when $u= 1$, the first term is active and the other is zero, and conversely, if $u=0$, the second term is active and the first is zero.

- **Equation 6.** This equation is similar to equation 4, but it was assumed that the female has a maximum egg storage capacity $C$:

$$e\left( t+1 \right)=r\left( e\left( t \right)-p\left( n,t \right) \right)+h(t)\wedge C$$

(6)

where the number of eggs in the next time step is the minimum $\wedge$between an egg balance term $r(e\left( t \right)-p\left( n,t \right))+h\left( t \right)$ and an upper threshold that denotes the maximum egg capacity represented by the symbol $C$.

- **Equation 7.** This equation combined equations 5 and 6; the maximum egg storage capacity is always greater than the reabsorption threshold $u\left( C˃u \right)$. Therefore, the egg production equation proposed was:

$$e\left( t+1 \right)=C\vee e\left( t \right)-p\left( n,t \right)+h\left( t \right)\vee r\left( e\left( t \right)-p\left( n,t \right) \right)+h(t)$$

(7)

where $\vee$ is the symbol that denotes the maximum of two functions, here $e(t+1)$ is the maximum of the three terms separated by that symbol.

The modules described above were combined into 48 Hidden Markov Models (HMM) [8], which were named according to the equations contained in the modules of functional response (*A*-*F*), and egg production (0-7). For example, the model *A*0 is represented by the type I functional response curve with unlimited egg production, and model *F*3 is the generalized type III functional response curve [5,6], but including the female experience, with the assumption that the female is synovigenic, emerges with an egg complement $e\left( t=0 \right)$, produces $h\left( t \right)$ eggs every day, and the egg production changes at a steady pace $g$.

**References**

1. Holling CS. Some characteristics of simple types of predation and parasitism. Can Entomol. 1959;91: 385–398. doi:10.4039/Ent91385-7

2. Bruzzone OA, Aguirre MB, Hill JG, Virla EG, Logarzo G. Revisiting the influence of learning in predator functional response, how it can lead to shapes different from type III. Ecol Evol. 2022;12: e8593. Available: https://doi.org/10.1002/ece3.8593

3. Bruzzone OA, Logarzo GA, Aguirre MB, Virla EG. Intra-host interspecific larval parasitoid competition solved using modelling and bayesian statistics. Ecol Modell. 2018;385: 114–123.

4. Aguirre MB, Bruzzone OA, Triapitsyn S V, Diaz-Soltero H, Hight SD, Logarzo GA. Influence of competition and intraguild predation between two candidate biocontrol parasitoids on their potential impact against Harrisia cactus mealybug, *Hypogeococcus* sp. (Hemiptera: Pseudococcidae). Sci Rep. 2021;11: 13377. doi:10.1038/s41598-021-92565-6

5. Real LA. The kinetics of functional response. Am Nat. 1977;111: 289–300.

6. Kalinkat G, Schneider FD, Digel C, Guill C, Rall BC, Brose U. Body masses, functional responses and predator-prey stability. Ecol Lett. 2013. doi:10.1111/ele.12147

7. Jervis MA, Heimpel GE, Ferns PN, Harvey JA, Kidd NAC. Life-history strategies in parasitoid wasps: a comparative analysis of ‘ovigeny’. J Anim Ecol. 2001;70: 442–458. doi:10.1046/j.1365-2656.2001.00507.x

8. Baum LE, Petrie T. Statistical inference for probabilistic functions of finite state Markov Chains. Ann Math Stat. 1966;37: 1554–1563. doi:10.1214/aoms/1177699147
